# Treatment monitoring by biomarker analysis in a Phase I dose-expansion study of AZD2811 for relapsed/refractory small-cell lung cancer

**DOI:** 10.1038/s41416-026-03414-0

**Published:** 2026-04-03

**Authors:** Melissa L. Johnson, Giulia Fabbri, Carmela Ciardullo, Judy S. Wang, Gerald S. Falchook, Suzanne Jones, Donald Strickland, Jacob Sands, Carl M. Gay, Robert J. Cardnell, Luis Tobalina, Sophie E. Willis, Jaime Rodriguez-Canales, Myria Nikolaou, Emma V. Jones, Stein Schalkwijk, Liz Sainsbury, Alexander MacDonald, Philip Overend, Caroline Kennedy, J. Elizabeth Pease, Philip Szekeres, Jan Cosaert, Howard Burris, Lauren A. Byers

**Affiliations:** 1 Sarah Cannon Research Institute, Nashville, TN USA; 2 Tennessee Oncology, Nashville, TN USA; 3 AstraZeneca, Waltham, MA USA; 4 AstraZeneca, Cambridge, UK; 5 Florida Cancer Specialists and Research Institute, Sarasota, FL USA; 6 Sarah Cannon Research Institute at HealthONE, Denver, CO USA; 7 Dana-Farber Cancer Institute, Boston, MA USA; 8 The University of Texas MD Anderson Cancer Center, Houston, TX USA; 9 AstraZeneca, Gaithersburg, MD USA

**Keywords:** Tumour biomarkers, Small-cell lung cancer

## Abstract

**Background:**

Aurora kinase B (AURKB) is overexpressed in lung cancer and is associated with poor prognosis. AZD2811 is an AURKB inhibitor that demonstrated tolerability during a Phase I dose-escalation study in patients with advanced solid tumours, including small-cell lung cancer (SCLC). Here we report the dose-expansion results.

**Methods:**

Eligible patients received nanoparticle-formulated AZD2811 500 mg IV (Day 1; 21-day cycles) with granulocyte colony-stimulating factor (Day 8). Dose-expansion endpoints included: preliminary antitumour activity, safety/tolerability, pharmacokinetics, and biomarker-based disease monitoring.

**Results:**

One of 21 enrolled patients achieved a partial response for an objective response rate of 4.8%; stable disease ≥6 weeks was observed in 10 patients (47.6%). The most common AZD2811-related AEs were decreased neutrophil and white blood cell count, anaemia, and decreased platelet count; grade ≥3 AZD2811-related AEs occurred in 15/21 patients. Baseline ctDNA levels were prognostic, and on-treatment ctDNA changes mirrored clinical response and identified progression early, suggesting it could be an effective surrogate for tumour tissue. Molecular profiling of paired tumour biopsies demonstrated AZD2811 pharmacodynamic activity and identified genes/pathways potentially linked to response.

**Conclusion:**

A personalised surveillance strategy may provide a novel avenue to monitor SCLC, supporting further investigation and potential broader clinical application.

**Clinical Trial Registration:**

NCT02579226

## Introduction

Small-cell lung cancer (SCLC) is a very aggressive disease characterised by rapid emergence of acquired resistance to platinum and a dismal outcome [1]. While SCLC has been historically viewed as a homogeneous and highly metastatic disease, recent investigations have revealed a previously unrecognised molecular and clinical diversity, with the identification of distinct molecular subtypes, largely defined by differential expression of key lineage-specific transcription factor genes (*ASCL1*, *NEUROD1*, and *POU2F3)*, or by an “Inflamed” gene signature [2–4]. This molecular characterisation has potential clinical implications, given preliminary observations of correlation with distinct therapeutic vulnerabilities in preclinical and clinical settings [3].

Genetic investigations of SCLC tumour samples have demonstrated that almost all cases have inactivation of the tumour suppressor genes *RB1* and *TP53* [5–7], which may explain the rapid and sustained proliferation that is characteristic of the disease. Since inactivation of *RB1* and *TP53* leads to accelerated mitosis, agents that target mitosis represent a rational option for the treatment of SCLC.

Aurora kinase B (AURKB) is a member of the family of serine/threonine kinases that have key roles in mitosis and chromosome segregation [8]; AURKB inhibition causes the failure of normal chromosome alignment and cytokinesis, resulting in polyploidy and apoptosis [9, 10]. AURKB is overexpressed in a variety of tumour types, including lung cancers, and is associated with poor prognosis and treatment resistance [10–13]; SCLC cells lacking the *RB1* tumour suppressor gene have been shown to be hyper-dependent on AURKB for survival [14].

AZD2811 is a highly potent and selective AURKB inhibitor. A prodrug of AZD2811, barasertib, showed promising efficacy in patients with acute myeloid leukaemia when administered as a continuous 7-day intravenous infusion [15], but in patients with advanced solid tumours, barasertib was frequently associated with bone marrow toxicities and had limited clinical activity [16]. A nanoparticle-encapsulated formulation of AZD2811 was subsequently developed to address the limitations of continuous infusion. Following a 2- to 4-hour infusion, slow release of AZD2811 from the nanoparticle maintains the concentration of free AZD2811 in plasma above the level required for disruption of tumour cell cytokinesis for a similar duration to the barasertib infusion administered over 7 days [17].

The nanoparticle formulation of AZD2811 was assessed in a Phase II study of Korean patients with recurrent SCLC for whom platinum-based chemotherapy had failed (SUKSES-N3). AZD2811, administered at a dose of 200 mg on Days 1 and 4 of 28-day cycles, showed early signs of clinical activity: there were no objective responses, but 4/15 (26.7%) patients had disease control >3 months [18].

This Phase I dose-escalation and expansion study (NCT02579226; REFMAL 390) also assessed the nanoparticle formulation of AZD2811. Results from the dose-escalation part of the study were reported previously: at the maximum tolerated dose/recommended Phase 2 dose (MTD/RP2D) of 500 mg on Day 1 of a 21-day cycle, with granulocyte colony-stimulating factor (G-CSF) administered on Day 8, AZD2811 was tolerable in patients with advanced solid tumours [19]. On-target neutropenia was a pharmacodynamic biomarker and was manageable with the addition of prophylactic G-CSF. Here we report clinical results from the expansion part of the study in patients with platinum-relapsed/refractory SCLC who received AZD2811 as second-line or later therapy. We also report exploratory biomarker data from paired tumour biopsies and longitudinal blood samples collected during the study. Obtaining adequate serial tumour biopsies beyond the initial diagnostic specimen is particularly challenging in SCLC, which has hampered much-needed understanding of disease biology and dynamic changes occurring on treatment. Liquid biopsies have the potential to be used as a rapid, readily applicable, and noninvasive alternative to tumour tissue to shed light on the molecular profile of the disease and to monitor its clonal evolution over time due to treatment pressure. However, circulating tumour DNA (ctDNA) datasets in this disease setting, especially in the context of novel therapies, are still limited [20–23].

## Results

### Demographics and baseline characteristics

Between December 18, 2018 and July 18, 2019, 21 patients were enrolled in the dose-expansion phase and received at least one dose of AZD2811. All patients had received previous treatment with standard-of-care platinum-etoposide; 11 had platinum-refractory disease (defined as relapse <3 months since the last line of platinum-based therapy) and 10 had platinum-relapsed disease (defined as relapse ≥3 months since the last line of platinum-based therapy). The data cutoff date was February 7, 2020. Patient demographics and disease characteristics are shown in Table 1 (representativeness of study participants is described in Supplementary Table 1). Median age was 65.0 years (range 52–74), 47.6% of patients were male, and 90.5% of patients were white. The median number of previous systemic therapies (chemotherapy, hormonal therapy, immunotherapy, targeted therapy, or radiation therapy) was 2.0 (range 1–3). Three patients received platinum-etoposide in combination with immune checkpoint inhibitors.

**Table 1:** Patient demographics and disease characteristics.

	*N* = 21
Age (years)	
Median (min, max)	65.0 (52, 74)
Sex (*n*, %)	
Female	11 (52.4)
Male	10 (47.6)
Race (*n*, %)	
White	19 (90.5)
Black or African American	1 (4.8)
Asian	1 (4.8)
ECOG performance status (*n*, %)	
0	6 (28.6)
1	15 (71.4)
Stage (*n*, %)	
IIIA	2 (9.5)
IIIB	3 (14.3)
IIIC	2 (9.5)
IV	13 (61.9)
IVA	0
IVB	1 (4.8)
Local or regional recurrence (*n*, %)	
Yes	10 (47.6)
No	11 (52.4)
Distant metastases (*n*, %)	
Yes	20 (95.2)
No^a^	1 (4.8)
Metastatic site (*n*, %)	
Bone	11 (52.4)
Brain	5 (23.8)
Distant lymph nodes	8 (38.1)
Liver	13 (61.9)
Local or regional lymph nodes	16 (76.2)
Lung	3 (14.3)
Other	11 (52.4)
Skin or subcutaneous	1 (4.8)
Albumin (g/L), median (min, max)	41.0 (30, 45)
Prior therapy (*n*, %)	
Radiotherapy – 0 session	4 (19.0)
Radiotherapy – 1 session	6 (28.6)
Radiotherapy – 2 sessions	7 (33.3)
Radiotherapy – ≥3 sessions	4 (19.0)
Systemic therapy – 1 line	10 (47.6)
Systemic therapy – 2 lines	7 (33.3)
Systemic therapy – 3 lines	4 (19.0)
Prior immunotherapy	
Yes	10 (47.6)
No	11 (52.4)
Chemotherapy-free interval	
<90 days	8 (38.1)
≥90 days	13 (61.9)

^a^Patient had subcarinal lymph node and right hilar lymph node involvement at baseline. ECOG, Eastern Cooperative Oncology Group.

### Safety

Patients received a median of 3 (range 1–17) cycles of treatment with AZD2811. The median duration of treatment was 64 days (range 19–377) (Supplementary Fig. 1) and the number of cycles showed no marked difference by number of prior systemic therapies, prior use of immunotherapy, or duration of chemotherapy-free interval (Supplementary Tables 2‒4).

All-cause adverse events (AEs) occurred in 100% of patients (Table 2); the most common were neutrophil count decreased (57.1%), anaemia (47.6%), aspartate aminotransferase increased (42.9%), hyperglycaemia (33.3%), and alanine aminotransferase increased (33.3%). Grade ≥3 all-cause AEs were reported in 20 patients (95.2%), most commonly neutrophil count decreased (57.1%), white blood cell count decreased (28.6%), neutropenia (19.0%), and hyponatraemia (19.0%). All-cause serious AEs (SAEs) occurred in 11 patients (52.4%); the most common were febrile neutropenia (14.3%) and neutropenia (9.5%).

**Table 2:** All-cause AEs occurring in ≥ 20% of patients (all grades), and grade ≥ 3 AEs occurring in ≥ 2 patients.

All-cause AEs, *n* (%)	*N* = 21	
	Total	Grade ≥ 3
Neutrophil count decreased	12 (57.1)	12 (57.1)
Anaemia	10 (47.6)	3 (14.3)
Aspartate aminotransferase increased	9 (42.9)	2 (9.5)
Hyperglycaemia	7 (33.3)	2 (9.5)
Alanine aminotransferase increased	7 (33.3)	1 (4.8)
White blood cell count decreased	6 (28.6)	6 (28.6)
Nausea	6 (28.6)	0
Platelet count decreased	5 (23.8)	3 (14.3)
Fatigue	5 (23.8)	1 (4.8)
Back pain	5 (23.8)	0
Diarrhoea	5 (23.8)	0
Hypokalaemia	5 (23.8)	3 (14.3)
Dyspnoea	5 (23.8)	1 (4.8)
Neutropenia	4 (19.0)	4 (19.0)
Hyponatraemia	4 (19.0)	4 (19.0)
Febrile neutropenia	3 (14.3)	3 (14.3)
Pneumonia	3 (14.3)	2 (9.5)
Confusional state	2 (9.5)	2 (9.5)

AE, adverse event.

Adverse events related to AZD2811 occurred in 19 patients (90.5%) (Supplementary Table 5). The most common were neutrophil count decreased (57.1%), white blood cell count decreased (28.6%), anaemia (28.6%) and platelet count decreased (23.8%). Grade ≥3 AZD2811-related AEs were reported in 15 patients (71.4%); the most common were neutrophil count decreased (57.1%), white blood cell count decreased (28.6%) and neutropenia (19.0%).

Serious AEs related to AZD2811 (per reporting investigator) occurred in 7 patients (33.3%): 1 patient with septic shock, 1 patient with neutropenia; 1 patient with anaemia; 1 patient with febrile neutropenia, streptococcal bacteraemia, and pneumonia; 1 patient with febrile neutropenia; 1 patient with nausea and diarrhoea; and 1 patient with febrile neutropenia, constipation, neutropenia, sinus tachycardia, confusional state, and hypotension.

Two patients died due to AEs; 1 death was related to AZD2811 (the SAE of septic shock described above), and the other (SAE of hypoxia) was unrelated to treatment and occurred after the 30-day follow-up.

Dose interruptions due to AEs occurred in 5 patients (23.8%); grade 2 nausea and grade 3 confusional state in 1 patient, grade 1 infusion-related reaction in 1 patient, grade 2 infusion-related reactions in 2 patients, and grade 2 confusional state, hypotension, and sinus tachycardia in 1 patient. Dose reductions due to AEs were required in 5 patients (23.8%). Three patients had their dose reduced to 400 mg (due to febrile neutropenia in 1 patient, maculopapular rash in 1 patient and neutropenia in 1 patient), 1 patient had a dose reduction to 400 mg then 300 mg (due to febrile neutropenia and neutropenia), and 1 patient had a dose reduction to 400 mg due to decreased platelet count and then 200 mg due to further toxicity (thrombocytopenia).

### Pharmacokinetics

At Cycle 1, the geometric mean area under the concentration-time profile from time of AZD2811 dose up to 504 hours post-dose was 8294 µg*h/mL (% coefficient of variation [CV] 65.9%), and the geometric mean maximum concentration was 115 µg/mL (%CV 26.7%). These parameters were consistent with those reported for the equivalent dose in the escalation phase of this study in patients with solid tumours [19].

### Preliminary antitumour activity and survival

**Fig. 1:**
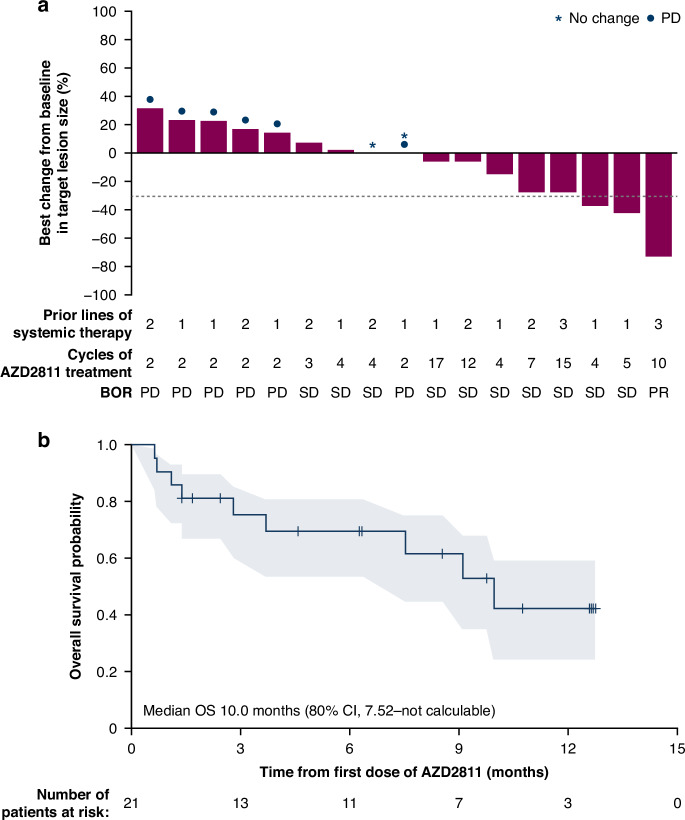
Preliminary antitumour activity and survival. **a** Best percentage change in target lesion size and **b** Kaplan-Meier estimate of overall survival. BOR best overall response, CI confidence interval, OS overall survival, PD progressive disease, PR partial response, SD stable disease.

One patient (4.8%) had a confirmed partial response (PR), and stable disease (SD) ≥ 6 weeks was observed in 10 patients (47.6%); of whom 2 (9.5% of the total population) had an unconfirmed complete response (CR) or PR (Supplementary Table 6 and Fig. 1a). Four patients (19.0%) had long stable disease (Long SD); their median duration of SD was 29.8 weeks (range: 14.7–50.7 weeks).

Median overall survival (OS) was 10.0 months (80% confidence interval [CI]: 7.52–not calculable) (Fig. 1b). With a median duration of follow-up of 6.24 months in all patients and 7.44 months in censored patients, the 6-month OS rate was 69.4% (80% CI: 53.6–80.7) and the 12-month OS rate was 42.3% (80% CI: 24.4–59.2).

### Evidence of pharmacodynamic activity of AZD2811

Paired tumour biopsies were obtained from 20/21 patients at baseline and on-treatment to analyse the pharmacodynamic effects of AURKB inhibition. In total, both pre- and on-treatment samples from 12 patients were evaluable by immunohistochemistry (IHC) for phospho-histone H3 (pHH3) and Cleaved Caspase 3 (CC3). Pharmacokinetic/pharmacodynamic modelling of previously published preclinical in vivo data (NCI-H417a SCLC xenograft [14]) indicated that the timing of the on-treatment biopsy (between 3 and 7 days post-infusion) was more suitable for detecting CC3 induction than pHH3 inhibition. Accordingly, CC3 was induced on treatment in most patients (7/12, 58.3%); biomarker modulation met the prespecified criteria in 5 patients, supporting the notion that AZD2811 sufficiently inhibits its target, leading to the desired downstream effect in relevant tumour tissue (Supplementary Fig. 2a). Conversely, pHH3 was reduced on treatment in 4/12 patients (33.3%), reflecting the expectedly low baseline levels of this marker and the suboptimal timing of the on-treatment biopsy for this marker (data not shown). Of note, the single patient with a PR displayed modulation of both pHH3 and CC3, meeting the prespecified criteria on therapy (Supplementary Fig. 2b and Materials).

These data indicate that AZD2811 has sufficient PK exposure and safety margin to demonstrate target engagement at a well-tolerated dose.

### ctDNA profiling recapitulated the genetic landscape of SCLC

To characterise the genetic landscape of patients with relapsed SCLC at baseline and monitor ctDNA dynamic changes over the course of AZD2811 treatment, ctDNA analysis was performed on 101 longitudinally collected blood samples from 20 patients (Supplementary Fig. 3a, b). Thirteen patients had matched germline DNA. Plasma cell-free DNA (cfDNA) was sequenced to a median depth of 6171× (range, 982×–15798×), with germline DNA sequenced to a median depth of 11108× (range, 566×–12409×).

**Fig. 2:**
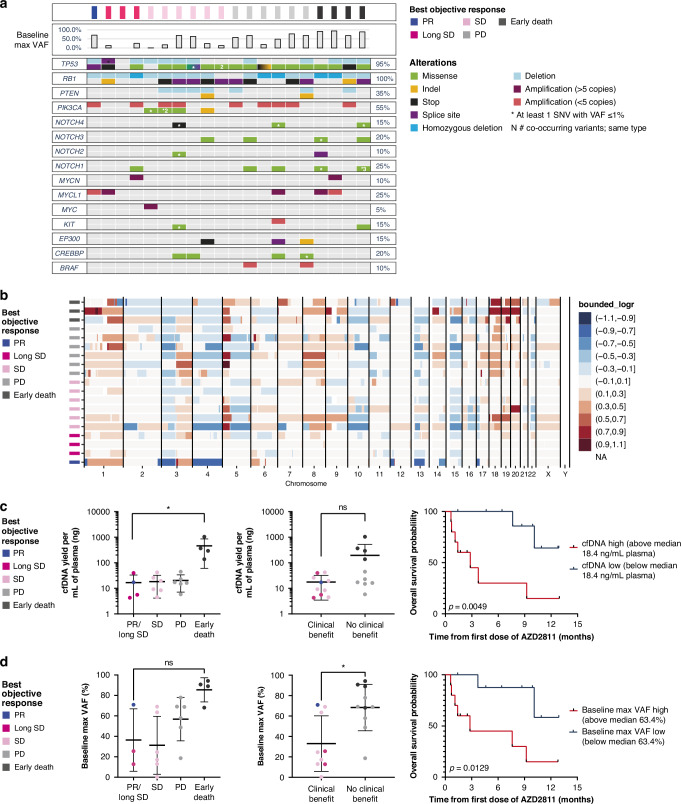
ctDNA profiling at baseline and relationship to clinical outcome. **a** Somatic mutation profiles of 20 patients from pretreatment ctDNA sequencing of 15 cancer genes. Patients’ identifiers are colour-coded by best objective response. Alterations are colour-coded per figure legend on the right-hand side of the image. The mutation frequencies for each gene are shown as percentages on the right panel of the oncoprint. The light-grey bar chart on top of the oncoprint indicates the VAF of the major mutated clone identified in pretreatment ctDNA samples. **b** Whole genome heatmap representing relative copy number profiles of the pretreatment samples. Segments are colour-coded according to their relative log2 copy number ratios, with regions in red indicating copy number gains and regions in blue indicating copy number losses. The x-axis represents each chromosome from 1 to 22. **c** Prognostic impact of baseline cfDNA yield. Left and centre: scatterplots of baseline cfDNA yield per mL of plasma by response status. Dots are colour-coded by the best objective response. Horizontal lines and error bars indicate mean ± SD. Right: Kaplan-Meier curves of overall survival for patients stratified by baseline cfDNA levels. **d** Prognostic impact of baseline maximum VAF. Left and centre: scatterplots of baseline maximum allele frequency by response status. Dots are colour-coded by the best objective response. Horizontal lines and error bars indicate mean ± SD. Right: Kaplan-Meier curves of OS for patients stratified by baseline maximum VAF. cfDNA cell-free DNA, ctDNA circulating tumour DNA, Long SD best objective response of SD and at least two post-baseline SD recorded, ns not significant, OS overall survival, PD progressive disease, PR partial response, SD stable disease, SNV single nucleotide variant, VAF variant allele frequency.

Disease-associated genetic lesions and copy number changes were identified in all patients, with a genetic landscape largely concordant with previous genetic investigations in both primary tumours and plasma samples [6, 7, 20]. Patients carried nearly universal disruption of *TP53* and *RB1*, and recurrent alterations in NOTCH family genes (*NOTCH1-4)*, PI3K pathway genes (*PIK3CA* copy number gains and *PTEN* disrupting events*)*, epigenetic regulators *(CREBBP* and *EP300)*, and amplifications of MYC family members (*MYC*, *MYCL1*, and *MYCN*, Fig. 2a). Similarly, recurrent gains (e.g., 3q and 5p) and losses (e.g,. 3p and 4) were found in the majority of cases (Fig. 2b), as previously reported [21]. Copy number aberrations detected by the targeted sequencing panel were orthogonally confirmed by low-pass whole genome (LPWG) sequencing; examples are shown in Supplementary Fig. 4 for *MYC* and *MYCL1* amplifications and *RB1* and *TP53* deletions. *TP53* variant allele frequency (VAF) was high (median 44.4% at Cycle 1 Day 1 and 18.3% over all visits; Supplementary Fig. 5a), consistent with the increased tumour shedding in SCLC as compared with other tumour types [20, 24].

Together, these results highlight the feasibility of using ctDNA as a non-invasive tool providing real-time genomic information in patients with relapsed SCLC.

### Baseline cfDNA and circulating tumour cells associated with shorter survival

To understand the potential prognostic value of liquid biopsy in this high-shed, rapidly progressing disease, we explored the relationship between circulating biomarkers and clinical outcome. Median baseline cfDNA and maximum VAF values were calculated (18.4 ng/mL of plasma and 63.4%, respectively) as benchmarks for categorising patient-level values as “high” or “low” (above or below median, respectively). Higher baseline cfDNA levels and maximum VAF identified nonresponding patients and/or patients in accelerated disease phase experiencing early death (i.e., before the first control scan at 6 weeks), and were consistently associated with shorter OS (log rank *p* = 0.005 for low vs high cfDNA; log rank *p* = 0.015 for low vs high VAF). Median OS was not reached for patients with low cfDNA and was 2.8 months for patients with high cfDNA; median OS was not reached for those with low VAF and was 2.8 months for those with high VAF (Fig. 2c, d).

Baseline cfDNA yield, and maximum and mean VAF did not correlate with tumour burden in this cohort of patients, as measured by the sum of target lesions (Supplementary Fig. 5b, c), similar to previous reports [22]. This may reflect the higher sensitivity of liquid biopsy approaches in capturing lesions compared to standard imaging technologies, providing additional complementary information. Likewise, baseline cfDNA yield, and maximum and mean VAF showed no association with gender, Eastern Cooperative Oncology Group performance status (ECOG PS), or presence of liver or lung metastases before treatment (Supplementary Fig. 6). Similarly, patients with higher circulating tumour cell (CTC) counts at baseline had shorter OS than those with lower counts, suggesting that CTCs also had prognostic significance (Supplementary Fig. 7a, b).

### Association of on-treatment ctDNA and CTC changes with clinical response

**Fig. 3:**
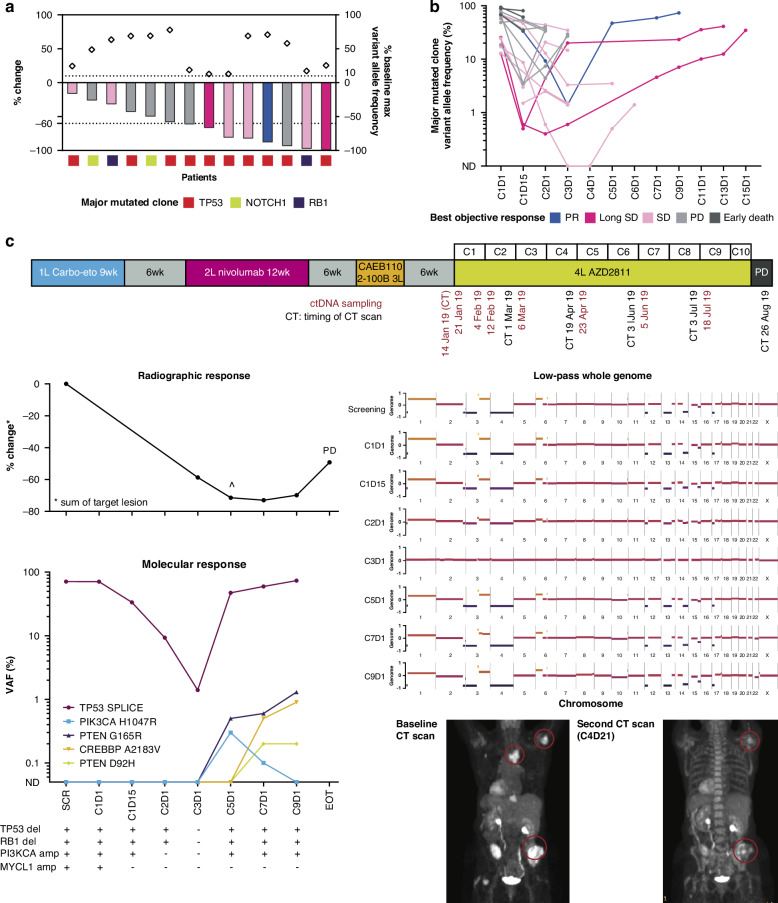
ctDNA dynamics during AZD2811 treatment. **a** Percentage change in major mutated clone’s VAF between pretreatment C1D1 and C2D1. Bars are colour-coded by best objective response. Major mutated clones are colour-coded by gene. Grey diamonds in upper panel show VAF of major mutated clone at baseline. Patients harbouring major mutations with VAF < 0.5% were excluded from the analysis. **b** On-treatment ctDNA dynamics of major mutated clones from baseline. Patients are colour-coded by best objective response. **c** Representative example of ctDNA dynamics in a patient who had a PR to AZD2811. The timeline shows the patient’s clinical course from first-line therapy to the date of progression on AZD2811. Percent mutant allele frequency and low-pass whole genome are shown below the timeline. The copy number alterations table indicates the presence or absence of the copy number alteration listed. Radiographic images (CT scans) obtained at baseline and at the end of Cycle 4 depicting shrinkage of tumour lesions are shown on the right. Carbo-eto carboplatin-etoposide, CT computed tomography, ctDNA circulating tumour DNA, EOT end of therapy, Long SD best objective response of SD and at least two post-baseline SD recorded, PD progressive disease, PR partial response, SD stable disease, VAF variant allele frequency, wk week. Therapy cycles are preceded by the letter ‘C’ and days of the cycle by the letter ‘D’.

Serial plasma samples from 20 patients were analysed throughout treatment to determine whether changes in ctDNA corresponded with clinical response. In patients who had a PR or SD, the VAF of the major clone (that with the highest VAF at baseline) markedly decreased after 1 treatment cycle (Fig. 3a, b), likely reflecting the decrease in tumour burden. In particular, a > 60% reduction in maximum VAF was observed in 6/8 (75.0%) patients who had a PR or SD, but only in 1/6 (16.7%) patients with progressive disease (PD) or early death. Of note, in this patient, the AZD2811 dose was reduced to 400 mg due to neutropenia at Cycle 2 Day 1, and progression was noted 3 weeks later.

**Fig. 4:**
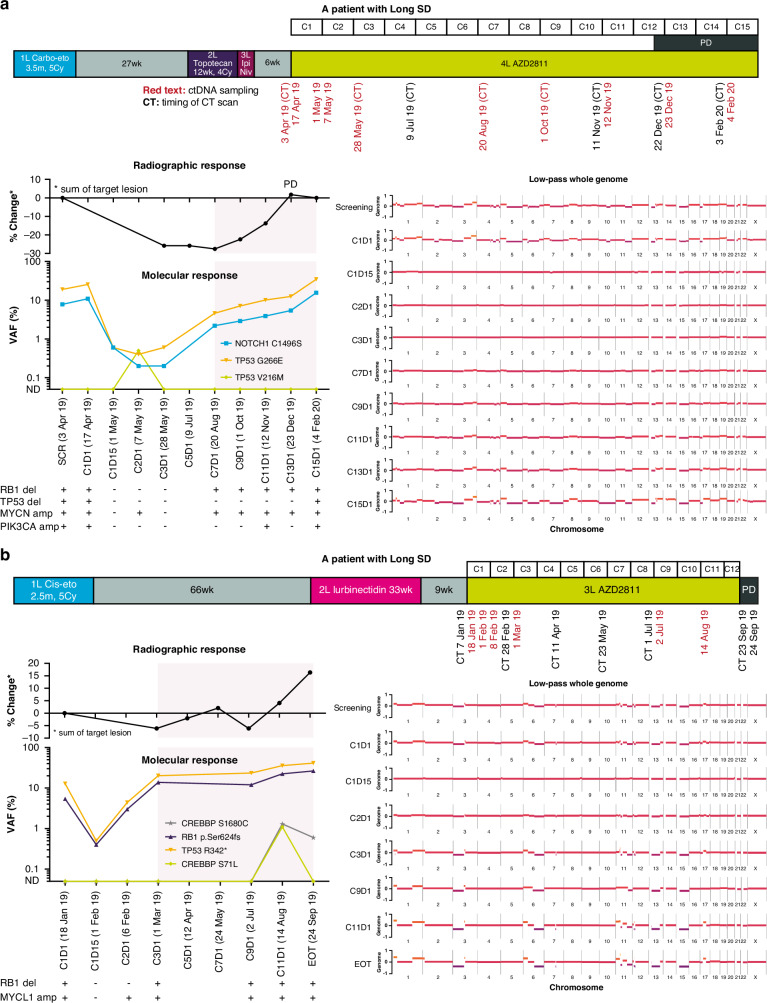
ctDNA anticipation of radiographic progression. **a** and **b** Representative examples of ctDNA dynamics in two patients who had Long SD while receiving AZD2811. The timelines show the patients’ clinical course from first-line therapy to the date of progression on AZD2811. Percent variant allele frequency and low-pass whole genome are shown below the timelines. The copy number alteration tables indicate the presence or absence of the copy number alteration listed. The light red area indicates the timeframe between molecular progression and radiographic progression. Carbo-eto carboplatin-etoposide, cis-eto cisplatin-etoposide, ctDNA circulating tumour DNA, Cy cycle, EOT end of therapy, Ipi ipilimumab, Long SD best objective response of SD and at least two post-baseline SD recorded, Niv nivolumab, PD progressive disease, SD stable disease, VAF variant allele frequency, wk week. Therapy cycles are preceded by the letter ‘C’ and days of the cycle by the letter ‘D’.

In several cases, longitudinal ctDNA analysis identified disease relapse or recurrence before the time of disease progression by conventional imaging, with mutated clones expanding up to 10 cycles before the time of overt relapse (Figs. 3c, 4 and Supplementary Fig. 8). The on-treatment genome-wide changes mirrored the ctDNA dynamics of the major mutated clones (Figs. 3c and 4), suggesting that both copy number and targeted analysis are effective means of disease monitoring.

In contrast, CTC dynamics did not mirror clinical response (median ~5.9 CTCs/mL [range: 0–165.6 CTCs/mL] at Cycle 1 Day 1; Supplementary Fig. 7c). These data indicate that CTC dynamics may be less powerful in predicting the trajectory of the disease than ctDNA due to lower sensitivity.

### Molecular profiling enabled subtype classification and provided insights into AZD2811 response

In order to classify patients into distinct subtypes and identify markers of response and resistance to AZD2811, the following additional analyses were performed: (a) the subtype-defining markers ASCL1, NEUROD1, and POU2F3, and additional proteins reported to be associated with sensitivity to Aurora kinase inhibitors and/or differentially expressed in distinct subtypes (MYC, BCL2, CDH1, VIM, AXL) were assessed by IHC in 38 biopsies (7 archival, 15 screening, and 16 post-treatment biopsies from 20 patients); (b) ASCL1, NEUROD1, and POU2F3 were assessed by RNA in situ hybridisation (ISH) analysis of 26 biopsies (6 archival, 9 screening, and 11 post-treatment biopsies from 18 patients); and (c) gene expression profiling was performed on 34 biopsies via a custom 800-gene Nanostring panel, including subtype-defining signatures and immune-related, cell cycle, and apoptotic family genes (4 archival, 15 screening, and 15 on-treatment biopsies from 15 patients). Subtype information was available both at baseline and on-treatment for 15/21 patients, only at baseline for 5/21 patients, and only on-treatment for one patient.

We observed a remarkable concordance between RNA-based and protein-based subtyping methods, with 18/20 cases being the ASCL1 subtype, 1 case the POU2F3 subtype and 1 case the Inflamed subtype at baseline (Supplementary Fig. 9a, b). The post-treatment biopsy of 1 patient who did not have a baseline biopsy belonged to the ASCL1 subtype (Supplementary Fig. 9a, b). Consistent with this observation, there was a notable correlation between mRNA and protein expression of analysed markers, and between expression of subtype-defining genes and of individual subtype-defining signatures (Supplementary Fig. 9c, d and data not shown).

Among patients with the ASCL1 subtype, 1 had a PR, and 8 had SD ≥ 6 weeks (including 4 with Long SD; Supplementary Fig. 9e, f).

### Subtype switching

The SCLC subtype identified in pretreatment specimens remained stable after treatment in most patients. However, 1 patient with SD switched from the POU2F3 subtype in the archival sample to the Inflamed subtype in the on-treatment biopsy (collected 266 days after archival biopsy collection; Supplementary Fig. 9a, b, d). Subtype evolution was accompanied by an increase in *MYC*, *VIM,* and *AXL* expression, hallmarks of the Inflamed subtype [2] (Supplementary Fig. 9a, 10a). Notably, this patient subsequently received immune checkpoint blockade therapy with a response lasting 1 year and 5 months. Another patient, who died early in the study, switched from the ASCL1 subtype at screening to the NEUROD1 subtype on treatment; the switch was accompanied by a reduction in *BCL2* expression (Supplementary Fig. 10a).

### Subtype heterogeneity

While IHC analysis indicated that the vast majority of analysed cases represented a single subtype, IHC H-scores highlighted that most tumours were likely composed of multiple cellular subpopulations expressing other subtype-defining transcription factors (Supplementary Fig. 10b). This observation is consistent with the hypothesis that the intratumour heterogeneity reported in SCLC reflects subtype plasticity that defines the natural history of the disease [2, 25, 26].

The expression of *ASCL1* correlated with that of its transcriptional target *BCL2* both at the mRNA and protein level (Supplementary Fig. 10c, d), in line with the known sensitivity of the ASCL1 subtype to BCL2 inhibitors [2, 27].

The expression of the MYC oncogene family members *MYC*, *MYCL,* and *MYCN* was largely mutually exclusive, with high mRNA levels being consistent with the presence of copy number gains identified via ctDNA analysis in some patients (e.g. *MYCL* high with high-level amplification in 2 patients, *MYC* high with high-level amplification in 1 patient, and *MYCN* high with high-level amplification in 1 patient; Supplementary Fig. 10e). *MYC* mRNA expression significantly correlated with that of *POU2F3* (*p* < 0.0001), consistent with previous findings [2] (Supplementary Fig. 10f). Despite the overall low MYC protein levels, MYC-high patients achieved a PR or SD with AZD2811 (1 patient with the Inflamed subtype carrying a high-level *MYC* amplification, and 1 patient with the POU2F3 subtype; Supplementary Fig. 10g). Of note, MYC-positive CTCs were detected only in these 2 patients, consistent with the findings in primary tumours (representative image in Supplementary Fig. 10h).

Together, these results suggest that most relapsed/refractory SCLC cases in this study had the ASCL1 subtype and confirm the molecular heterogeneity of SCLC at the single patient level.

### Gene expression features associated with response

To investigate transcriptional features related to treatment response and detect changes occurring on treatment, we deployed a custom Nanostring panel encompassing genes related to SCLC subtype, immune function, cell cycle regulation, apoptosis, Aurora kinase inhibitor sensitivity, and housekeeping genes (*n* = 800).

Gene expression profiling of immune-related genes, including a previously published 18-gene IFNγ-related T-cell signature [28], showed that most patients had a baseline transcriptional landscape consistent with ‘cold’ tumours, characterised by poor immune-cell infiltration and low expression of IFN signatures, immune checkpoint genes, and human leucocyte antigen (HLA) genes (Supplementary Fig. 11a), in line with previous reports [2]. Notably, the 1 patient on study who had a PR as best overall response showed a baseline transcriptional profile consistent with a ‘hot’ phenotype. Although the interval between baseline and on-treatment biopsy in this cohort was likely too short to detect significant changes in immune infiltration during AZD2811 treatment, this patient also displayed upregulation of immune markers in the tumour biopsy (e.g., CTLA4 and CXCL9; Supplementary Fig. 11b) and several inflammatory cytokines in the periphery (e.g,. IP-10, CXCL9, RANTES, MIP1a, IL-12, TNF-α, and IL-2; Supplementary Fig. 11c) after treatment. This supports the notion that AZD2811 could, in an unknown proportion of patients, contribute to antitumour immunity, which may result in combination efficacy with PD-L1 blockade [29].

To identify baseline features associated with response to treatment, we compared the expression profiles of baseline tumour samples of patients with either a PR or SD to those of patients with PD or early death (3 and 4 eligible samples, respectively). Screening samples obtained from PR and SD patients showed higher expression of several neuroendocrine-related genes (e.g. *SCG2*, *SCN3A*, *TFF3*) and some immune-related genes (e.g. *HLA-G*, *HLA-K*) compared with those obtained from patients with PD or early death. The same comparison with post-treatment samples (5 PR or SD samples vs 7 PD or early death samples) also highlighted higher expression of *SCG2*, *SCN3A*, *TTF3,* and *HLA-G* in patients who had a PR or SD (Supplementary Fig. 12a). Notably, a patient-by-patient analysis in search of genes that showed significantly different expression compared with the overall cohort identified high expression of *CCND1* and very low expression of *CDKN2A*, *CCNE1,* and *SOX1* in 1 patient with Long SD (Supplementary Fig. 12b).

Evaluable (i.e., ≥20% of tumour content) paired samples were available for 3 patients with PR or SD and 4 patients with PD or early death. Differential gene expression between screening and on-treatment samples revealed modest changes in gene expression (generally effect sizes <1.5 log-fold), with a few genes, such as *CALB2* and *KLHL13*, showing upregulation, and *IRF4* showing downregulation on AZD2811 treatment in patients with PR or SD. A modest upregulation of *NEUROD2* was observed in patients with PD or early death (log2-fold change, 0.945; *p* = 0.0185; Supplementary Fig. 13).

Overall, gene expression analysis showed the potential to uncover determinants of having a PR or SD or resistance, suggesting that further investigations of gene expression are needed in future studies.

## Discussion

In the dose-expansion part of this Phase I trial, over half of patients with SCLC receiving AZD2811 as second- or later-line treatment achieved a PR or SD; 1 patient (4.8%) had a PR and 10 patients (48%) experienced SD ≥ 6 weeks. Notably, the median OS in this study was 10.0 months (80% confidence interval: 7.52–not calculable); however, our study is single-arm, small, with a relatively short follow-up of patients, and not powered for median OS, therefore, the data should be interpreted with caution. In addition, AZD2811 500 mg Q3W with mandatory G-CSF support (the RP2D identified in the dose-escalation part of the study) [19] was found to have a manageable safety profile overall. The AE profile, comprising predominantly bone marrow and gastrointestinal AEs, was consistent with that observed in the dose-escalation part of the study [19], and in other Phase I studies assessing Aurora kinase inhibitors in advanced solid tumours [16, 30–32]. In particular, the frequent on-target decreases in neutrophil count were generally manageable and associated with low treatment discontinuation rates.

Target engagement was documented in paired tumour specimens, with induction of CC3, a marker of apoptosis, in most patients (58.3%), supporting the hypothesis that AZD2811 sufficiently inhibited its target, leading to the desired effect in tumour tissue.

One of the key exploratory findings of this study is the relevance of blood-based profiling as a promising novel avenue to monitor patients with SCLC. ctDNA analysis detected disease-associated mutations and copy number changes in all patients, with VAFs consistent with SCLC being a highly shedding disease, in line with its very aggressive nature [24]. The patterns and frequencies of detected genomic alterations were largely concordant with previous genetic investigations obtained by large-scale tissue sequencing data and in plasma samples [6, 7, 20–22], supporting the feasibility of using ctDNA genomic profiling as a powerful surrogate for tumour tissue. In addition to nearly universal *TP53* and *RB1* disruptions, several genes were recurrently altered in this cohort, including NOTCH, MYC, and PI3K pathway family members and epigenetic regulators. These observations highlight distinct molecular mechanisms and functionally relevant biological programs that are likely to be important for the pathogenesis and therapeutic targeting of the disease. Notably, we found an increased representation of alterations in PI3K pathway genes (e.g., *PTEN* 35%, *PIK3CA* 55%) and MYC family genes (e.g., *MYC* 5%, *MYCN* 10%, *MYCL1* 25%), compared to studies enriched with treatment-naïve patients [5, 6, 19–22]. Our findings in this relapsed/refractory SCLC cohort support previous observations that correlate MYC family activity with an aggressive, drug-resistant phenotype, and PI3K pathway activation with fast disease kinetics [33, 34].

From a clinical perspective, baseline ctDNA burden, as documented by higher cfDNA levels and VAF, was significantly higher in nonresponding patients and/or patients in the accelerated disease phase experiencing early death, and it predicted shorter OS in this study. Notably, higher ctDNA levels at baseline did not correlate with other clinical parameters such as tumour burden as assessed by conventional radiographic imaging, gender, ECOG PS, or presence of liver or lung metastases before treatment. This suggests the potential superiority of liquid biopsy in correctly capturing the heterogeneity, complexity, and disseminated nature of this aggressive malignancy. It also highlights a potential limitation of RECIST assessment in SCLC, as rapid tumour growth and metastasis typically result in high tumour burden by disseminated and/or confluent lesions (i.e., non-target lesions not contributing to the sum of target lesions but leading to high ctDNA levels).

Similarly, higher baseline levels of CTCs predicted shorter OS in this cohort, suggesting that both methodologies could potentially be used in a clinically meaningful timeframe to identify patients requiring further intervention, such as those with tumours in an accelerated growth phase, who may need to start treatment quickly.

The clinical relevance of ctDNA monitoring in SCLC was further strengthened by the observation that early ctDNA dynamics correlated with clinical response, with the greatest reductions in VAF within 1 cycle being observed in patients who achieved a PR or SD and likely reflecting the decrease in tumour burden. In addition, longitudinal monitoring of both mutations and copy number aberrations enabled early detection of disease recurrence before radiographic progression. Similar ctDNA findings were reported in the Phase II TAZMAN study of durvalumab plus etoposide and carboplatin or cisplatin for first-line treatment of patients with extensive-stage SCLC: early VAF reductions were associated with chemosensitivity and, in several cases, ctDNA changes anticipated disease relapse before conventional imaging [35]. While further studies will provide a better estimate of the interval between molecular and clinical progression (‘ctDNA lead time’), these data support the potential clinical utility of serial ctDNA monitoring to predict treatment response and anticipate radiographic progression. This, in turn, could translate into early salvage intervention and prolonged benefits. An approach that is noninvasive and easily repeatable over time is appealing as obtaining adequate tumour biopsies is very challenging in this disease setting due to inaccessible anatomical locations, comorbidities, and insufficient tissue quantity and/or quality (i.e., frequent necrosis and crush artifacts).

Overall, our findings support the systematic inclusion of molecular blood-based surveillance in SCLC clinical studies, and highlight its potential to improve the management of this disease at multiple levels, including early detection, molecular profiling, monitoring of response, minimal residual disease detection, anticipation of progression, and understanding of clonal evolution and mechanisms of resistance.

Although noninvasive approaches are needed, especially for longitudinal disease monitoring, this study confirmed the utility of collecting research biopsies for exploratory analyses in the context of a controlled clinical study with the goal of understanding the biological bases of disease and drug action. In addition to demonstrating pharmacodynamic activity, the longitudinal tumour samples were profiled using multidimensional approaches, including gene expression (Nanostring and RNA-ISH) and IHC. The timing of on-treatment biopsy collection was selected to maximise detection of AZD2811 pharmacodynamic effects (particularly CC3), while balancing patient safety and comfort. This approach may limit the ability to assess long-term changes in subtype classification and other molecular features.

To our knowledge, this is the first dataset in which SCLC tumour biopsies were systematically subtyped utilising both RNA-based and protein-based methods, and revealing good concordance. Tissue-based subtyping approaches have the potential to be further developed for standard diagnostic workup and to inform treatment decisions.

Most patients enrolled in this study had the ASCL1 subtype, resulting in a higher observed prevalence compared to previously published datasets. However, direct comparisons with previous studies are limited by both the small size of our cohort, which precludes drawing definitive conclusions, and differences in molecular subtyping methodology, as our study employed a Nanostring-based RNA platform rather than RNA sequencing.

As previously reported [2, 4], our investigation supports the notion that relapsed SCLC is a heterogenous disease at the individual level, with coexpression of multiple subtype-defining markers in the same tumour specimen. This is consistent with the hypothesis that intratumour heterogeneity plays a role in the natural history of the disease [2–4, 25, 26, 36]. While dramatic changes in the dominant subtype were not observed after a few days of AZD2811 treatment, 1 patient who switched from the POU2F3 subtype in the archival biopsy to the Inflamed subtype in the on-treatment biopsy (collected 266 days after archival biopsy collection) had a durable response of almost 1.5 years on subsequent immunotherapy, in line with the reported sensitivity of Inflamed tumours to immune checkpoint inhibitors [2]. These findings, while limited to a single case, suggest that pharmacologic induction of an inflammatory transcriptional state, regardless of the specific agent, may also potentially represent a viable strategy to enhance the efficacy of emerging immunotherapies, including bispecific T-cell engagers, in SCLC.

Another patient, who died before the first scan on AZD2811 therapy, had a dramatic switching of subtype within 6 days of treatment (ASCL1 to NEUROD1), suggesting rapid clonal escape on therapy or potentially reflecting intrapatient subtype heterogeneity.

Molecular analyses of subtype-related markers confirmed previously reported correlations with subtype-defining genes and proteins, specifically between BCL2 and ASCL1, and between MYC and POU2F3 [2, 37]. Similarly, mesenchymal proteins were higher in the one patient with the Inflamed subtype in this cohort, highlighting their potential utility as confirmatory markers of the Inflamed subtype in the clinic. Of note, high MYC levels were previously reported as potential markers of sensitivity to Aurora kinase inhibition in SCLC [33]. The 2 patients in this cohort who expressed high levels of MYC, documented both transcriptionally and via IHC analysis in tumour tissue and confirmed in CTCs, experienced disease stabilisation on AZD2811 treatment. Mechanistically, in 1 case, high-level amplification of the *MYC* locus was detected via ctDNA analysis, and in the other, MYC levels increased upon subtype switching from the archival to the on-treatment sample (H-score increase from 43 to 157). These observations are consistent with in vivo data suggesting that this oncogene drives subtype plasticity and cell fate reprogramming toward a non-neuroendocrine state in this disease [26].

Gene expression profiling of tumour tissues provided insights into the immune landscape of the disease, confirming the overall cold immune phenotype, with low expression of T-cell inflamed signatures. This analysis highlighted the potential of exploring AZD2811 as an immune priming agent, as previously suggested [29], to potentiate response to immune-based therapy, as demonstrated by 1 patient with PR showing increased inflammatory markers both in the tumour and in the periphery on therapy. Grouped and patient-by-patient analyses highlighted genes potentially associated with response, including neuroendocrine markers, immune-related genes, and high expression of Cyclin D1 in the context of low expression of CDKN2A, consistent with Aurora kinase hyperdependence in rapidly proliferating tumours due to cell cycle deregulation [14].

Although this study suggests that AZD2811 is a potentially effective and tolerable regimen for SCLC, clinical development has been discontinued. The study provided evidence of the high potential value of liquid biopsy as an effective means of baseline analysis and longitudinal monitoring of patients throughout treatment. Gene expression profiling of tumour tissue has provided insights into disease biology and a rationale for possible combinations, and has identified genes potentially linked to efficacy. A personalised surveillance strategy based on these findings may provide the earliest measure of treatment response and allow for earlier detection of relapse, thus improving overall outcomes for patients with platinum-relapsed/refractory SCLC. Given the small size of the analysed cohort, this monitoring strategy will require confirmation in broader studies.

## Materials And Methods

### Experimental design

This was a first-time-in-patient, multicentre, open-label study conducted in the US, consisting of a dose-escalation phase in patients with solid tumours and a dose-expansion phase in patients with SCLC, evaluating preliminary efficacy and exploring pharmacokinetics and biological activity. Detailed methods for the overall study have been described previously [19].

The expansion phase enrolled patients aged ≥18 years with SCLC, either platinum-relapsed disease (defined as relapse ≥3 months since the last line of platinum-based chemotherapy) or platinum-resistant disease (defined as relapse <3 months since the last line of platinum-based therapy). Patients must have received 1–3 prior lines of therapy for relapsed/refractory disease, and must not have previously relapsed during the first 4 cycles of platinum-based therapy. Patients were required to have an ECOG PS of 0 or 1, in addition to adequate bone marrow, liver, and renal function.

In the dose-escalation part of the study, the RP2D of AZD2811 was identified as 500 mg IV on Day 1 of each 21-day cycle, with mandatory G-CSF on Day 8 to limit the severity and duration of neutropenia and to minimise the risk of neutropenic fever and infections [19]. This RP2D was used in the dose-expansion part of the study to further characterise safety, preliminary efficacy, pharmacokinetics, and biological activity. Treatment continued until disease progression, unacceptable AEs, or fulfilment of other discontinuation criteria.

Tumour imaging assessments using computed tomography or magnetic resonance imaging were performed at screening, at the end of Cycle 2, then every 2 cycles for up to 1 year. Survival follow-up assessments were performed every 12 weeks for 1 year. AEs were assessed throughout treatment and follow-up periods. Patients were followed until resolution of all treatment-related toxicity, or for at least 30 days after study drug discontinuation. AEs were graded according to Common Terminology Criteria for Adverse Events (CTCAE) version 4.03. Venous blood samples (4 mL) were collected throughout Cycle 1 on Days 1, 4, 8, 12 and 15 to assess pharmacokinetics. Blood samples were collected as follows: at screening, on Days 1 and 4 of Cycle 1, then on Day 1 of each subsequent cycle, and at end of treatment for analysis of CTCs; at screening, on Days 1 and 15 of Cycles 1 and 2, then on Day 1 of each subsequent cycle for immunophenotyping; at screening, on Days 1, 4, 8 and 15 of Cycle 1, and on Day 1 of Cycle 2 for pharmacodynamic analyses; at screening, on Days 1 and 15 of Cycle 1, Day 1 of Cycle 2, then Day 1 of every subsequent odd-numbered cycle, and upon disease progression, for analysis of ctDNA. Mandatory paired tumour biopsies for molecular profiling and subtype analysis, gene expression profiling, and analysis of pharmacodynamic effects of AURKB inhibition were collected at screening and from the same lesion between Cycle 1 Day 4 and Day 6.

The primary endpoints for this study have been previously reported [19]. Here we report secondary efficacy endpoints for the dose-expansion phase: preliminary antitumour activity (objective response rate [ORR] based on RECIST version 1.1) and OS rate at 6 months. Other secondary endpoints were safety/tolerability, and pharmacokinetics (derived from AZD2811 total blood concentrations). Exploratory endpoints included biomarker analyses of tumour and blood/plasma samples, and Long SD, which was defined as a best objective response of SD and at least two post-baseline SD recorded.

### Statistical analysis

The planned sample size of 21 patients was sufficient to demonstrate that the study treatment exceeded a 10% ORR with 80% probability if the response rate was at least 19% (i.e., 4/21 responders) and to demonstrate that the survival rate exceeded 40% at 6 months. Continuous futility monitoring of ORR began after approximately 50% of patients were enrolled in the cohort, and allowed termination of the cohort for predicted lack of efficacy.

The safety analysis set comprised all patients who received at least one dose of AZD2811. The pharmacokinetic analysis set comprised all patients who received at least one dose of AZD2811 with at least one reportable concentration. The evaluable for efficacy analysis set comprised all patients who received at least one dose of AZD2811 and had a baseline tumour assessment. Safety and efficacy data were not formally analysed. The numbers of patients experiencing each AE were summarised by Medical Dictionary for Regulatory Activities (MedDRA) version 22.1 system organ class, MedDRA preferred term, and CTCAE grade. The ORR and best objective response based on RECIST version 1.1 were summarised. AZD2811 blood concentrations were summarised by nominal sample time.

### Biomarker analysis

#### Collection of tumour biopsies

Paired tumour biopsies, obtained as formalin-fixed paraffin-embedded (FFPE) blocks, were obtained at baseline (within 7–28 days of Cycle 1 Day 1) and on treatment (between Cycle 1 Day 4 and Day 6, 72–120 hours post-dosing). When available, historical archival tumour blocks or slides were also collected.

#### IHC and image analysis

IHC was performed on FFPE biopsy sections (3 μm), using pHH3 (Millipore #06-570, final concentration 0.75 μg/mL diluted in Leica Bond Diluent Leica SLBV2987), CC3 (CST #9661, final concentration 0.1 µg/mL diluted in DAKO Antibody Diluent DAKO S0809).

Sections were dewaxed, rehydrated, antigen retrieved, and stained on Leica Bond RX (pHH3), VENTANA Benchmark Discovery Ultra™ (CC3). Antigen retrieval used heat-induced epitope retrieval (HIER), ER1 (pHH3) or Standard Cell conditioning #1 at 95°C (CC3). Detection of primary antibodies was performed using Leica Bond Polymer Refine Detection Kit (DS9800) for pHH3, or Chromomap DAB Kit (Roche 760-159) for CC3.

Slides were scanned at x20 using the Aperio AT2 scanner (Leica Biosystems). Quantification of percent positive pHH3 tumour cells was performed using HALO (Indica Labs) CytoNuclear v1.5 image analysis algorithm. CC3 positivity was assessed using the HALO (Indica Labs) Area Quantification v1.0 algorithm.

Quantitative interpretation of CC3 induction upon treatment was achieved via translational/clinical PK/PD modelling of previously published in vivo data using the NCI-H417a SCLC xenograft model [14].

#### Cell-free DNA (cfDNA) analysis

Double-spun plasma was isolated from whole blood (10 mL) collected in Streck cell-free DNA BCT tubes at various timepoints before and during treatment. cfDNA was extracted from ~3 mL of plasma using standard methods and quantified using a Qubit fluorometer (Thermo Fisher). Up to 100 ng of cfDNA was used for whole-genome library construction. Each library was then split and subjected to LPWG sequencing and targeted enrichment using a custom panel of 15 genes (ctDx SCLC, Resolution Bioscience; Supplementary Fig. 3a). The custom panel was an optimised version of a previously reported gene panel [20] and was designed to detect substitutions, indels and copy number alterations in the coding region of 12 genes (*BRAF*, *CREBBP*, *EP300*, *KIT*, *NOTCH1*, *NOTCH2* coding exons 5–34, *NOTCH3*, *NOTCH4*, *PIK3CA*, *PTEN*, *RB1*, and *TP53)* using Resolution Bioscience’s bias-corrected targeted hybrid capture technology, as previously described [38]. The panel also contained probes for the detection of amplifications in the genes *MYC*, *MYCL1*, and *MYCN*, and control probes that targeted selected regions in all 22 autosomes. DNA from peripheral blood mononuclear cells of the same patient was sequenced using the same custom panel and served as the germline control to aid identification of clonal hematopoiesis of indeterminate potential (CHIP) and private germline variants [39, 40] (Supplementary Fig. 3b). Baseline cfDNA yield was quantified with the Qubit fluorometer and reported as ng/mL of plasma used for extraction. Baseline Max VAF was the maximum of VAF values among all somatic variants identified at cycle 1 day 1. Baseline Mean VAF was the average of VAF values among all the somatic variants identified at Cycle 1, Day 1.

#### CTC analysis

Blood samples (Streck cell-free DNA BCT tubes) were collected at multiple time points. At EPIC Sciences (San Diego, CA), after RBC lysis of blood, nucleated cells (3 × 10^6^) were deposited on glass slides. Cells that were CK + C45- and had an intact nucleus were classified as traditional CTCs. Additional CTC subtypes, CK- CTC clusters and apoptotic CTCs were also counted. CTC enumeration was normalised to blood volume and expressed as number detected per mL.

#### Gene Expression and RNA-ISH Analysis

To investigate transcriptomic characteristics related to treatment response, mRNA was extracted from FFPE pre- and post-treatment biopsy samples using the RNeasy FFPE extraction kit (Qiagen) and quantified using a Qubit High Sensitivity Kit (Thermo Fisher Scientific). 100 ng of RNA (or the maximum possible) was run on NanoString using an 800-gene bespoke codeset (21-hour hybridisation, 555 field of view). This custom NanoString panel encompassed genes related to SCLC subtyping, immune function, cell cycle regulation, apoptosis, and Aurora kinase inhibitor sensitivity, in addition to housekeeping genes. Log2 normalised gene expression data were generated after both positive control and housekeeping gene normalisation, and the background threshold was set at negative control +2 standard deviations through nSolver (NanoString technologies).

Differential gene expression analysis was performed using the limma package in R. Genes with outlier expression for each sample were identified as those with more than 1 absolute fold change and more than 2 standard deviations away from the mean expression of the gene across all samples and subsequently filtered through manual inspection. Samples with <20% tumour content as reported by hematoxylin and eosin staining were excluded during the analysis, but included in the figures.

RNA-ISH (RNAScope) was quantified using HALO image analysis (Indica Labs). Average probe copies per μm^2^ were assessed for each of the 4 probes. Subtypes were selected using the biomarker with the highest average probe copies per μm^2^.

## Supplementary information

Supplementary Materials

## Data Availability

Data underlying the findings described in this manuscript may be obtained in accordance with AstraZeneca’s data sharing policy described at https://astrazenecagrouptrials.pharmacm.com/ST/Submission/Disclosure. Data for studies directly listed on Vivli can be requested through Vivli at www.vivli.org. Data for studies not listed on Vivli could be requested through Vivli at https://vivli.org/members/enquiries-about-studies-not-listed-on-the-vivli-platform/. The AstraZeneca Vivli member page is also available, outlining further details: https://vivli.org/ourmember/astrazeneca/.
