# Supplementary material for: Treatment monitoring by biomarker analysis in a Phase I dose-expansion study of AZD2811 for relapsed/refractory small-cell lung cancer
Source: Br J Cancer. 2026 Apr 3;134(11):1592–604. doi: 10.1038/s41416-026-03414-0 (PMC13184323; doi:10.1038/s41416-026-03414-0)
Supplement: Supplementary file 1 — Supplementary Materials [file 41416_2026_3414_MOESM1_ESM.docx]

**SUPPLEMENTARY APPENDIX**

**Supplementary Table 1. Representativeness of study participants**

| **Cancer type(s) / subtype(s) / stage(s) / condition** | Platinum relapsed/refractory, stage IIIA–IVB small-cell lung cancer (SCLC) |
| --- | --- |
| **Considerations related to:** | |
| **Sex** | Historically, the incidence of SCLC was greater in males than females (10.9 per 100,000 vs 7.6 per 100,000, respectively, in the US in 2000) (1), but the incidence in males has declined over the last two decades, and the male:female ratio is now around 1:1 (2). In 2019, the incidence rates in males and females were 5.6 and 5.1 per 100,000, respectively (1). In a recent retrospective analysis of the SEER database, 50.7% of 26,221 patients diagnosed with SCLC between 2010 and 2015 were male (3). |
| **Age** | Patients with SCLC have a median age of approximately 65 years (4, 5); over 50% of 26,221 patients in the SEER database diagnosed with SCLC between 2010 and 2015 were ≥65 years old (3). The average age reported in two large studies is 60–70 years (6, 7); the peak of SCLC incidence occurs at age 75–79 years in both men and women (8). |
| **Race/ethnicity** | SCLC is more commonly observed in White people than those of other ethnicities (2). Of 26,221 patients diagnosed with SCLC in the US between 2010 and 2015, 82% were White (3). Black women are twice as likely as black men to develop SCLC (9). In the last two decades, rates of SCLC have declined across all races in the US (1). |
| **Geography** | In 2023, an estimated 238,340 adults will be diagnosed with lung cancer in the US (10). SCLC accounts for approximately 14% of all lung cancer cases (8). Approximately 30,000–35,000 people are diagnosed with SCLC in the US each year (9). |
| **Other considerations** | Patients with SCLC typically present with advanced disease, with only approximately 5% diagnosed at an early stage (stage I–II) (3).  In almost all cases, SCLC is attributable to cigarette smoking, which has been identified as the most important risk factor in SCLC tumorigenesis (6,11).  No significant differences in survival were observed between White and Black patients with SCLC treated between 1983 and 2012 (2). Median survival is approximately 4–8 months (2, 7). |
| **Overall representativeness of this study** | It should be acknowledged that this was a small study (N=21), which impacts the potential representativeness of the SCLC population.  The median age of patients in our study (65 years) is consistent with reports in the literature (4, 5).  The ratio of males to females is also aligned with recent reports (1, 2), with almost equivalent numbers of men (n=10; 48%) and women (n=11; 52%).  The vast majority (90.5%) of patients in our study were White, with 5% Black/African American and 5% of Asian origin. This is reflective of real-world observations: 82% of 26,221 SCLC patients in the US SEER database were White (3).  Most patients in our study had advanced disease (67% stage IV), which is also consistent with the 71% of patients with stage IV disease identified in the SEER database (3).  Our study does not report patient smoking history. |

SCLC, small-cell lung cancer

**References:**

# 1. National Cancer Institute. Surveillance, Epidemiology, and End Results Program. Available at: https://seer.cancer.gov/statistics-network/explorer/application.html. Accessed December 18, 2025.

# 2. Wang S, Tang J, Sun T, Zheng X, Li J, Sun H, Zhou X, Zhou C, Zhang H, Cheng Z, Ma H, Sun H. Survival changes in patients with small cell lung cancer and disparities between different sexes, socioeconomic statuses and ages. *Sci Rep* 2017;7:1339.

# 3. Arriola E, Trigo JM, Sánchez-Gastaldo A, Navarro A, Perez C, Crama L, Ponce-Aix S. Prognostic value of clinical staging according to TNM in patients with SCLC: A real-world Surveillance Epidemiology and End-Results database analysis. *JTO Clin Res Rep* 2021;3:100266.

# 4. Gao H, Dang Y, Qi T, Huang S, Zhang X. Mining prognostic factors of extensive-stage small-cell lung cancer patients using nomogram model. *Medicine (Baltimore)* 2020;99:e21798.

5. Ni J, Zhang X, Wang H, Si X, Xu Y, Zhao J, Chen M, Zhang L, Wang M. Clinical characteristics and prognostic model for extensive-stage small cell lung cancer: A retrospective study over an 8-year period. *Thorac Cancer* 2022;13:539–48.

# 6. Huang LL, Hu XS, Wang Y, Li JL, Wang HY, Liu P, Xu JP, He XH, Hao XZ, Jiang PD, Liu YT, Luo J, Zhou SY, Wang JW, Yang JL, Qin Y, Yuan P, Lin L, Shi YK. Survival and pretreatment prognostic factors for extensive-stage small cell lung cancer: A comprehensive analysis of 358 patients. *Thorac Cancer* 2021;12:1943–51.

# 7. O'Sullivan DE, Cheung WY, Syed IA, Moldaver D, Shanahan MK, Bebb DG, Sit C, Brenner DR, Boyne DJ. Real-world treatment patterns, clinical outcomes, and health care resource utilization in extensive-stage small cell lung cancer in Canada. *Curr Oncol* 2021;28:3091–103.

# 8. American Cancer Society. Cancer Facts and Figures 2023. Special section: Lung cancer. Available at: https://www.cancer.org/content/dam/cancer-org/research/cancer-facts-and-statistics/annual-cancer-facts-and-figures/2023/2023-cff-special-section-lung-cancer.pdf. Accessed December 18, 2025.

9. Ellis RR. WebMD. Who gets small-cell lung cancer? Available at: https://www.webmd.com/lung-cancer/who-gets-small-cell-lung-cancer. Accessed March 7, 2023.

# 10. Siegel RL, Miller KD, Wagle NS, Jemal A. Cancer statistics, 2023. *CA Cancer J Clin* 2023;73:17–48.

# 11. Ruano-Raviña A, Provencio-Pulla M, Pérez-Ríos M. Small cell lung cancer – a neglected disease with more data needed. *JAMA Netw Open* 2022;5:e224837.

**Supplementary Table 2. Number of AZD2811 cycles by prior lines of therapy**

| **Number of cycles** | **Overall** | **1 prior line of therapy** | **2–3 prior lines of therapy** |
| --- | --- | --- | --- |
| N | 21 | 10 | 11 |
| Median (min, max) | 3 (1, 17) | 3 (1, 17) | 3 (1, 15) |
| Number of cycles, n (%) |  |  |  |
| ≥1 cycles | 21 (100) | 10 (100) | 11 (100) |
| ≥2 cycles | 17 (81.0) | 9 (90.0) | 8 (72.7) |
| ≥3 cycles | 11 (52.4) | 5 (50.0) | 6 (54.5) |
| ≥4 cycles | 10 (47.6) | 5 (50.0) | 5 (45.5) |
| ≥5 cycles | 6 (28.6) | 2 (20.0) | 4 (36.4) |
| ≥6 cycles | 5 (23.8) | 1 (10.0) | 4 (36.4) |
| ≥7 cycles | 5 (23.8) | 1 (10.0) | 4 (36.4) |
| ≥8 cycles | 4 (19.0) | 1 (10.0) | 3 (27.3) |
| ≥9 cycles | 4 (19.0) | 1 (10.0) | 3 (27.3) |
| ≥10 cycles | 4 (19.0) | 1 (10.0) | 3 (27.3) |
| ≥11 cycles | 3 (14.3) | 1 (10.0) | 2 (18.2) |
| ≥12 cycles | 3 (14.3) | 1 (10.0) | 2 (18.2) |
| ≥13 cycles | 2 (9.5) | 1 (10.0) | 1 (9.1) |
| ≥14 cycles | 2 (9.5) | 1 (10.0) | 1 (9.1) |
| ≥15 cycles | 2 (9.5) | 1 (10.0) | 1 (9.1) |
| ≥16 cycles | 1 (4.8) | 1 (10.0) | 0 |
| 17 cycles | 1 (4.8) | 1 (10.0) | 0 |

**Supplementary Table 3. Number of AZD2811 cycles by prior immunotherapy**

| **Number of cycles** | **Overall** | **Prior immunotherapy** | **No prior immunotherapy** |
| --- | --- | --- | --- |
| N | 21 | 10 | 11 |
| Median (min, max) | 3 (1, 17) | 2.5 (1, 15) | 4.0 (1, 17) |
| Number of cycles, n (%) |  |  |  |
| ≥1 cycles | 21 (100) | 10 (100) | 11 (100) |
| ≥2 cycles | 17 (81.0) | 6 (60.0) | 11 (100) |
| ≥3 cycles | 11 (52.4) | 5 (50.0) | 6 (54.5) |
| ≥4 cycles | 10 (47.6) | 4 (40.0) | 6 (54.5) |
| ≥5 cycles | 6 (28.6) | 2 (20.0) | 4 (36.4) |
| ≥6 cycles | 5 (23.8) | 2 (20.0) | 3 (27.3) |
| ≥7 cycles | 5 (23.8) | 2 (20.0) | 3 (27.3) |
| ≥8 cycles | 4 (19.0) | 2 (20.0) | 2 (18.2) |
| ≥9 cycles | 4 (19.0) | 2 (20.0) | 2 (18.2) |
| ≥10 cycles | 4 (19.0) | 2 (20.0) | 2 (18.2) |
| ≥11 cycles | 3 (14.3) | 1 (10.0) | 2 (18.2) |
| ≥12 cycles | 3 (14.3) | 1 (10.0) | 2 (18.2) |
| ≥13 cycles | 2 (9.5) | 1 (10.0) | 1 (9.1) |
| ≥14 cycles | 2 (9.5) | 1 (10.0) | 1 (9.1) |
| ≥15 cycles | 2 (9.5) | 1 (10.0) | 1 (9.1) |
| ≥16 cycles | 1 (4.8) | 0 | 1 (9.1) |
| 17 cycles | 1 (4.8) | 0 | 1 (9.1) |

**Supplementary Table 4. Number of cycles by chemotherapy-free interval**

| **Number of cycles** | **Overall** | **Chemotherapy-free interval <90 days** | **Chemotherapy-free interval** **≥90 days** |
| --- | --- | --- | --- |
| N | 21 | 8 | 13 |
| Median (min, max) | 3 (1, 17) | 3.5 (1, 17) | 2.0 (1, 15) |
| Number of cycles, n (%) |  |  |  |
| ≥1 cycle | 21 (100) | 8 (100) | 13 (100) |
| ≥2 cycles | 17 (81.0) | 5 (62.5) | 12 (92.3) |
| ≥3 cycles | 11 (52.4) | 5 (62.5) | 6 (46.2) |
| ≥4 cycles | 10 (47.6) | 4 (5.0) | 6 (46.2) |
| ≥5 cycles | 6 (28.6) | 2 (25.0) | 4 (30.8) |
| ≥6 cycles | 5 (23.8) | 2 (25.0) | 3 (23.1) |
| ≥7 cycles | 5 (23.8) | 2 (25.0) | 3 (23.1) |
| ≥8 cycles | 4 (19.0) | 2 (25.0) | 2 (15.4) |
| ≥9 cycles | 4 (19.0) | 2 (25.0) | 2 (15.4) |
| ≥10 cycles | 4 (19.0) | 2 (25.0) | 2 (15.4) |
| ≥11 cycles | 3 (14.3) | 1 (12.5) | 2 (15.4) |
| ≥12 cycles | 3 (14.3) | 1 (12.5) | 2 (15.4) |
| ≥13 cycles | 2 (9.5) | 1 (12.5) | 1 (7.7) |
| ≥14 cycles | 2 (9.5) | 1 (12.5) | 1 (7.7) |
| ≥15 cycles | 2 (9.5) | 1 (12.5) | 1 (7.7) |
| ≥16 cycles | 1 (4.8) | 1 (12.5) | 0 |
| 17 cycles | 1 (4.8) | 1 (12.5) | 0 |

**Supplementary Table 5. Treatment-related AEs occurring in ≥5% of patients (any grade) and grade ≥3 treatment-related AEs occurring in ≥2 patients**

| **Treatment-related AEs, n (%)** | **N=21** | |
| --- | --- | --- |
|  | **Total** | **Grade ≥3** |
| Neutrophil count decreased | 12 (57.1) | 12 (57.1) |
| White blood cell count decreased | 6 (28.6) | 6 (28.6) |
| Anemia | 6 (28.6) | 2 (9.5) |
| Platelet count decreased | 5 (23.8) | 3 (14.3) |
| Neutropenia | 4 (19.0) | 4 (19.0) |
| Nausea | 4 (19.0) | 0 |
| Febrile neutropenia | 3 (14.3) | 3 (14.3) |
| Rash maculo-papular | 3 (14.3) | 1 (4.8) |
| Diarrhea | 3 (14.3) | 0 |
| Infusion-related reaction | 3 (14.3) | 0 |
| Vomiting | 3 (14.3) | 0 |
| Candida infection | 2 (9.5) | 0 |
| Stomatitis | 2 (9.5) | 0 |
| Fatigue | 2 (9.5) | 1 (4.8) |
| Rash | 2 (9.5) | 0 |
| Decreased appetite | 2 (9.5) | 0 |

AE, adverse event.

**Supplementary Table 6. Response by RECIST**

| **Response, n (%)** | **N=21** |
| --- | --- |
| Objective response rate  (80% CI)^a^ | 1 (4.8)  (0.5–17.3) |
| Best objective response^b^  Complete response  Partial response | 1 (4.8)  0  1 (4.8) |
| Non-response  Stable disease ≥6 weeks  Unconfirmed complete or partial response^c^  Progression  RECIST progression  Death | 20 (95.2)  10 (47.6)  2 (9.5)  10 (47.6)  6 (28.6)  4 (19.0) |
| Objective response or stable disease^d^  (80% CI) | 11 (52.4)  (36.4–68.0) |
| ^a^Exact two-sided Clopper-Pearson confidence interval.  ^b^Response required confirmation after 4 weeks.  ^c^Complete or partial response was observed but either no confirmation assessment was performed or a confirmation assessment was performed but response was not confirmed.  ^d^Complete or partial response or stable disease ≥6 weeks; post-hoc analysis.  CI, confidence interval; RECIST, Response Evaluation Criteria in Solid Tumors. | |

**Supplementary Figure 1. Time on AZD2811 therapy**


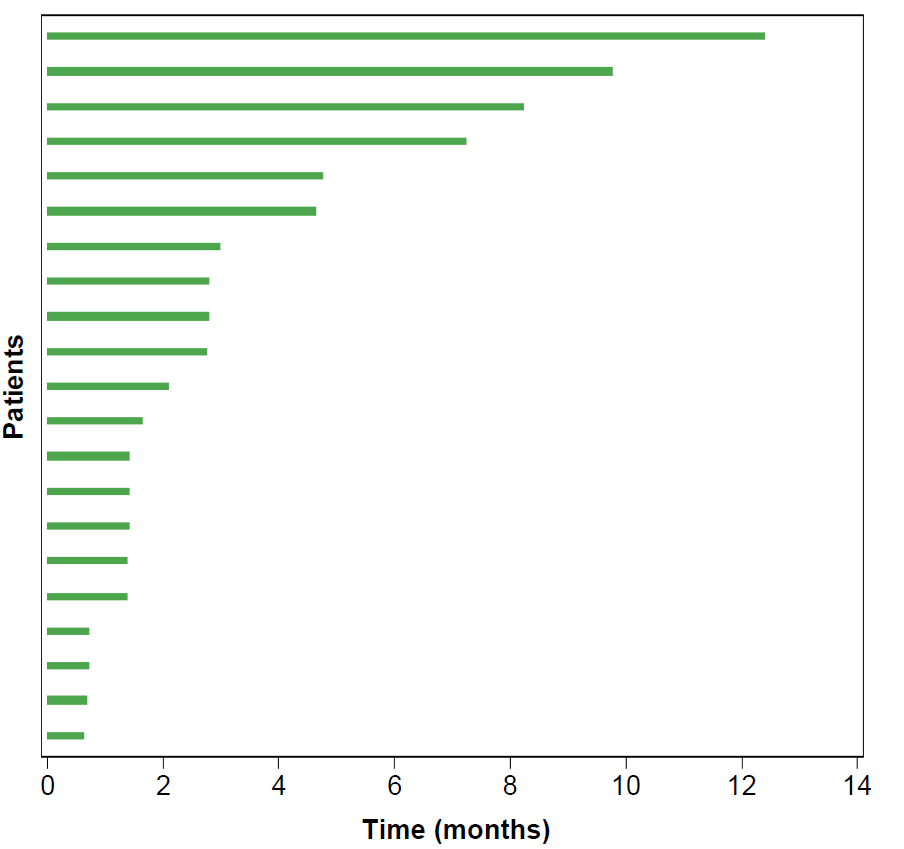


Swimmer plot depicting the time on AZD2811 therapy of individual SCLC patients included in the study.
SCLC, small-cell lung cancer.

**Supplementary Figure 2. Pharmacodynamic activity of AZD2811**


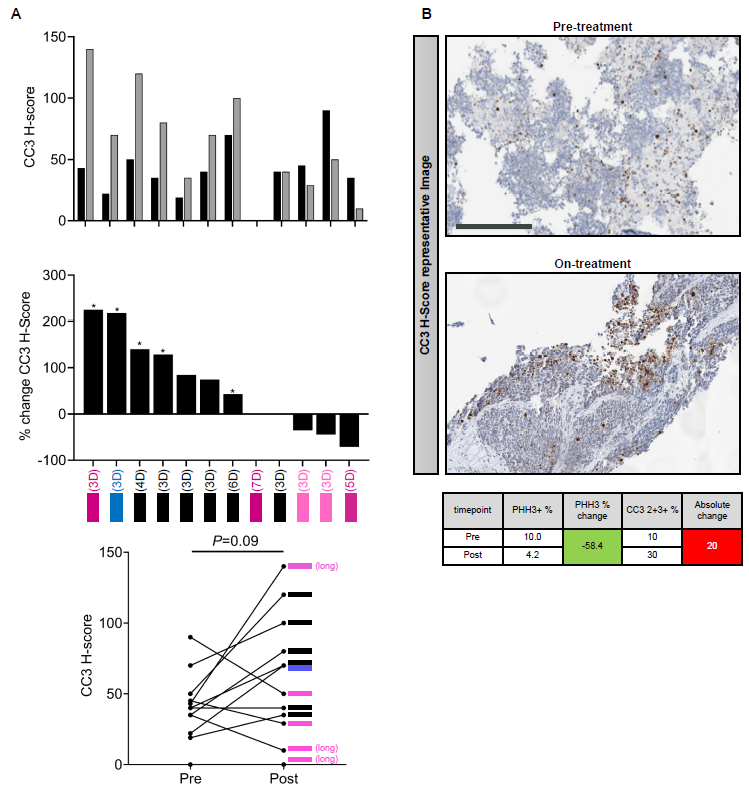


1. CC3 H-score (top and bottom) and % change (middle) between pre-treatment (black) and post-treatment (gray) tumor biopsies as assessed via IHC. Patients are color-coded based on their response to treatment (black, progressive disease or early death, light pink, stable disease; dark pink, Long SD; and blue, partial response). In the middle graph the interval in days (‘D’) between the pre- and post-treatment samples is reported in parentheses after the patient ID. *Cases with changes meeting the prespecified criteria.
2. CC3 and pHH3 IHC analysis of representative pre- and post-treatment tumor samples obtained in a patient with a partial response to AZD2811 treatment.

CC3, Cleaved Caspase 3; IHC, immunohistochemistry; Long SD, best objective response of SD and at least two post-baseline SD recorded; pHH3, phospho-histone H3.

**Supplementary Figure 3. Targeted and whole-genome NGS analysis in SCLC**


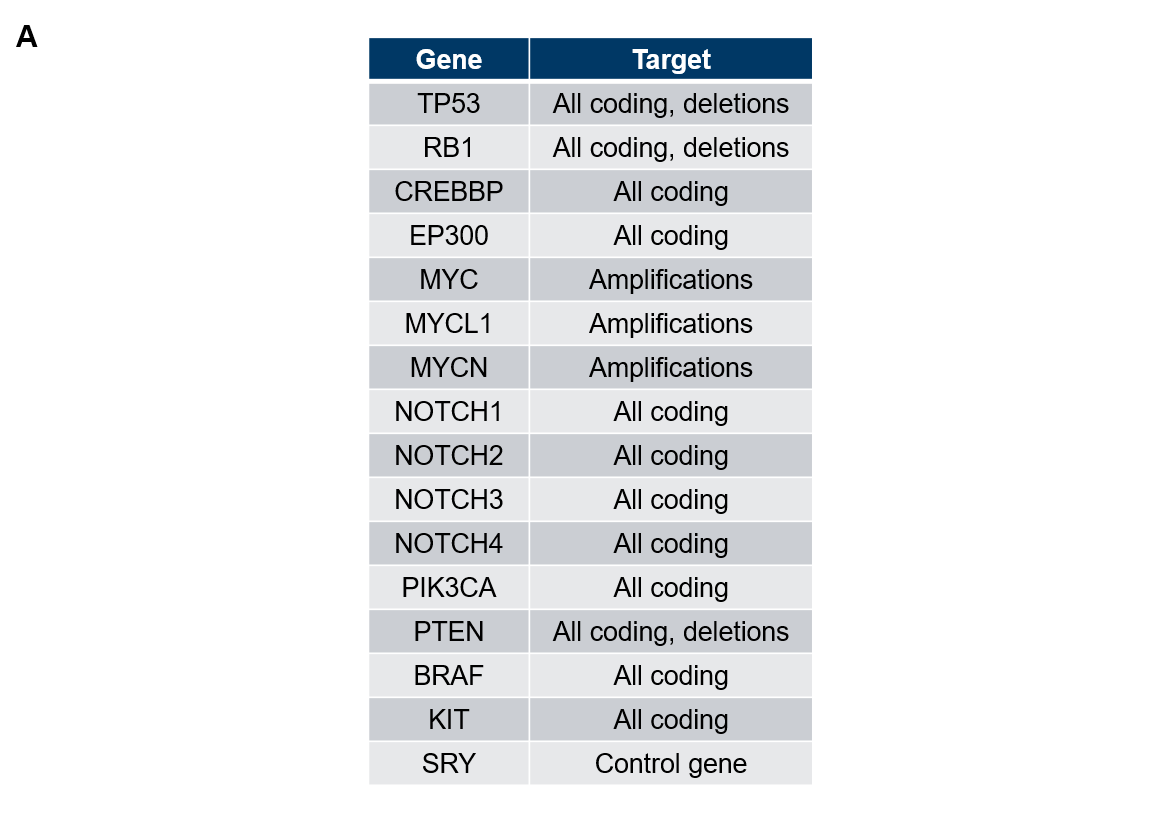


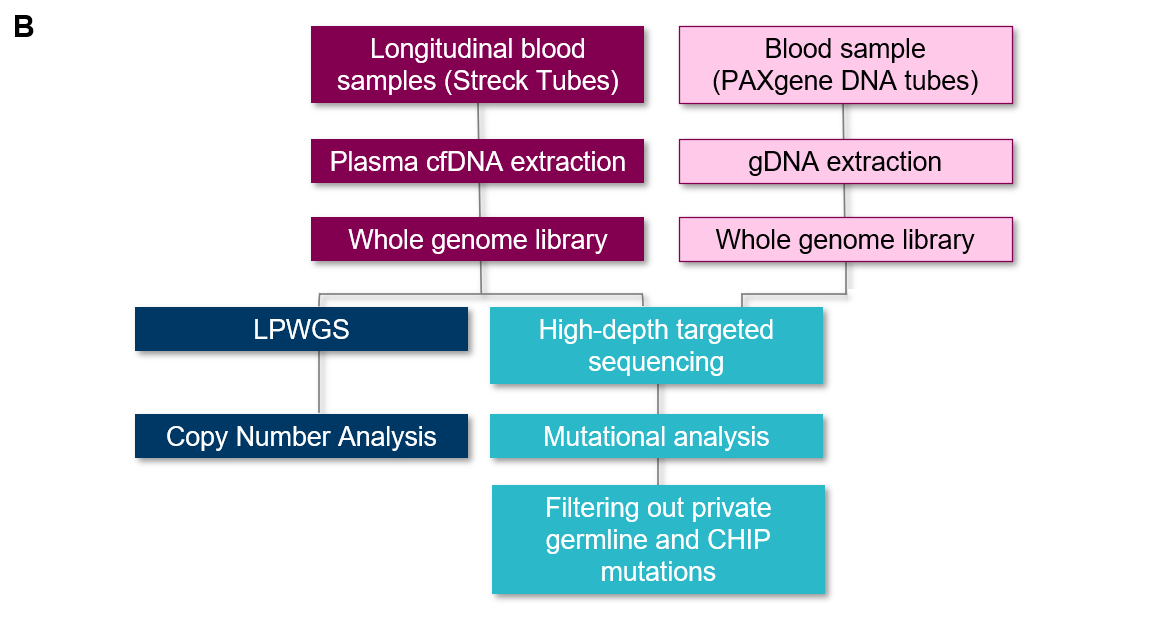


1. Resolution Bioscience’s ctDx SCLC panel targeting all coding exons of *BRAF, KIT, NOTCH1, NOTCH2, NOTCH3, NOTCH4, PIK3CA, CREBBP, EP300, PTEN, RB1,* and *TP53*. The panel also contains probes for detection of copy number amplifications in the genes *MYC, MYCL1,* and *MYCN*.
2. Workflow illustrating genomic profiling of SCLC ctDNA and whole blood.

CHIP, clonal hematopoiesis of indeterminate potential; cfDNA, cell-free DNA; ctDNA, circulating tumor DNA; gDNA, genomic DNA; LPWGS, low-pass whole genome sequencing; NGS, next-generation sequencing; SCLC, small-cell lung cancer.

**Supplementary Figure 4.** **Orthogonal validation of copy number aberrations identified in ctDNA by targeted sequencing and LPWGS**


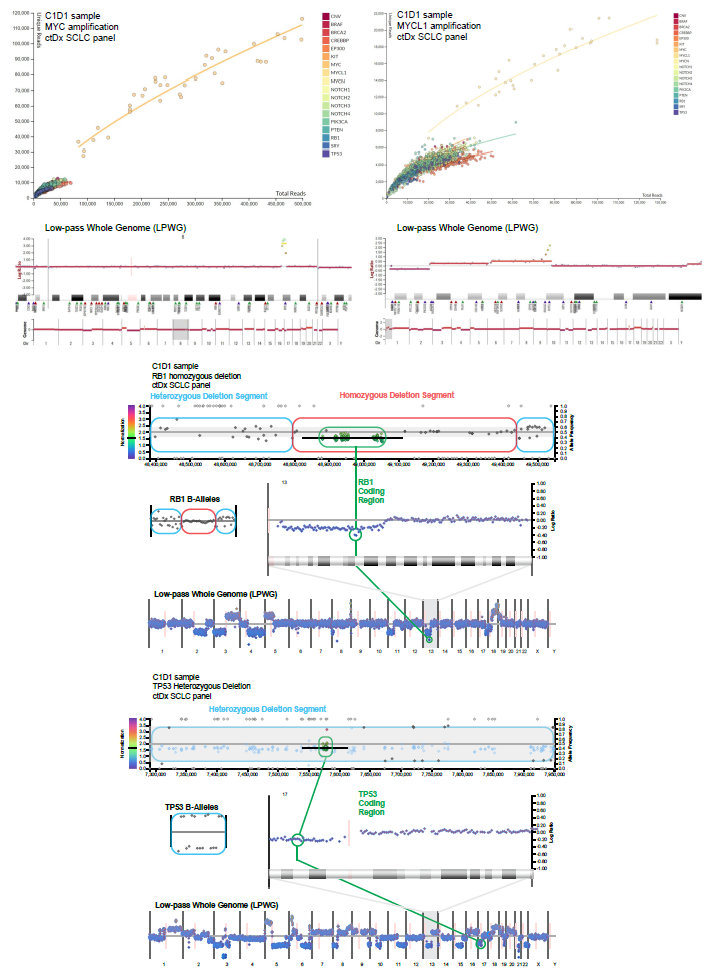


Representative examples of copy number amplification (top two panels) and copy number loss (bottom two panels) identified by targeted sequencing with ctDx SCLC panel and LPWGS

ctDNA, circulating tumor DNA; LPWGS; low-pass whole genome sequencing; SCLC; small-cell lung cancer. Therapy cycles are preceded by the letter ‘C’ and days of the cycle by the letter ‘D’.

**Supplementary Figure 5. Distribution of *TP53* VAF and correlation between baseline tumor burden and cfDNA levels, maximum and mean VAF**


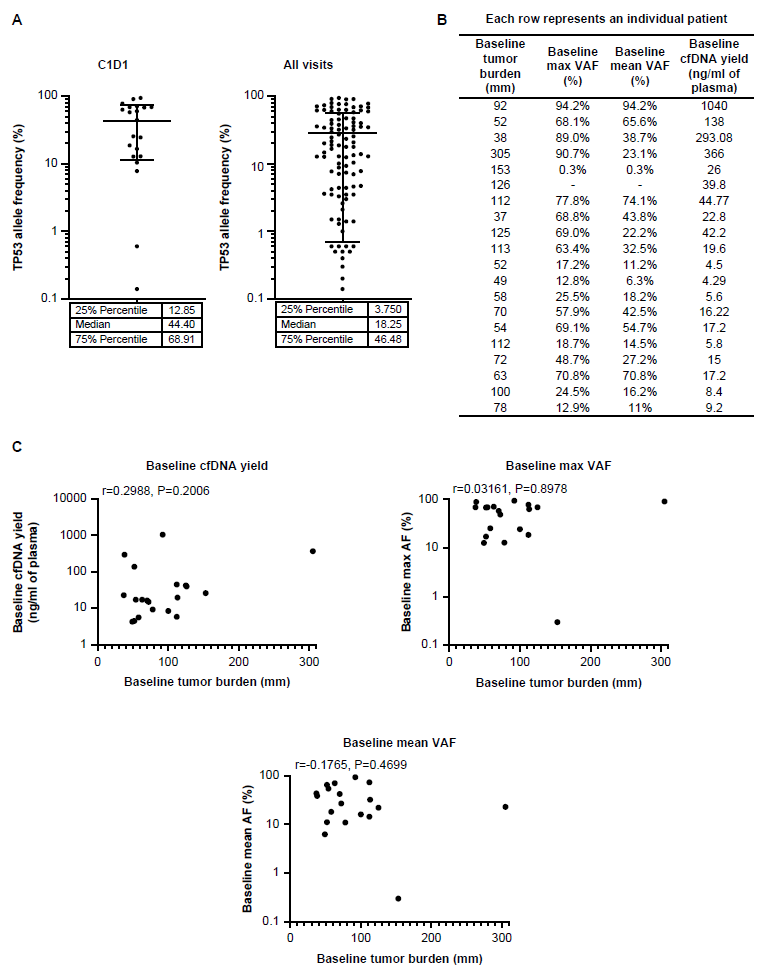


1. Scatterplot of VAF (%) of *TP53* mutations in our SCLC patient cohort at pretreatment C1D1 (left) and across all visits (right). Each individual patient who had a somatic *TP53* mutation is represented by a dot. Horizontal lines and error bars indicate mean ± standard deviation.
2. Baseline tumor burden (sum of target lesions in mm), maximum VAF (%), mean VAF (%) and cfDNA yield (measured as ng/mL of plasma used for extraction) across the entire patient cohort available for ctDNA longitudinal profiling.
3. Spearman correlation of baseline tumor burden and cfDNA levels, maximum and mean VAF. Tumor burden shown as sum of the target lesions on CT scans in mm. cfDNA levels shown as ng/mL of plasma used for extraction. Maximum VAF shown as percentage. Mean allele frequency shown as percentage. All parameters refer to measurements before any treatment. Spearman r and p-value are shown.

cfDNA, cell-free DNA; CT, computed tomography; ctDNA, circulating tumor DNA; SCLC, small-cell lung cancer; VAF, variant allele frequency. Therapy cycles are preceded by the letter ‘C’ and days of the cycle by the letter ‘D’.

**Supplementary Figure 6. Relationship of baseline cfDNA levels and maximum and mean VAF to gender, ECOG performance status, and presence of liver or lung metastases before treatment**


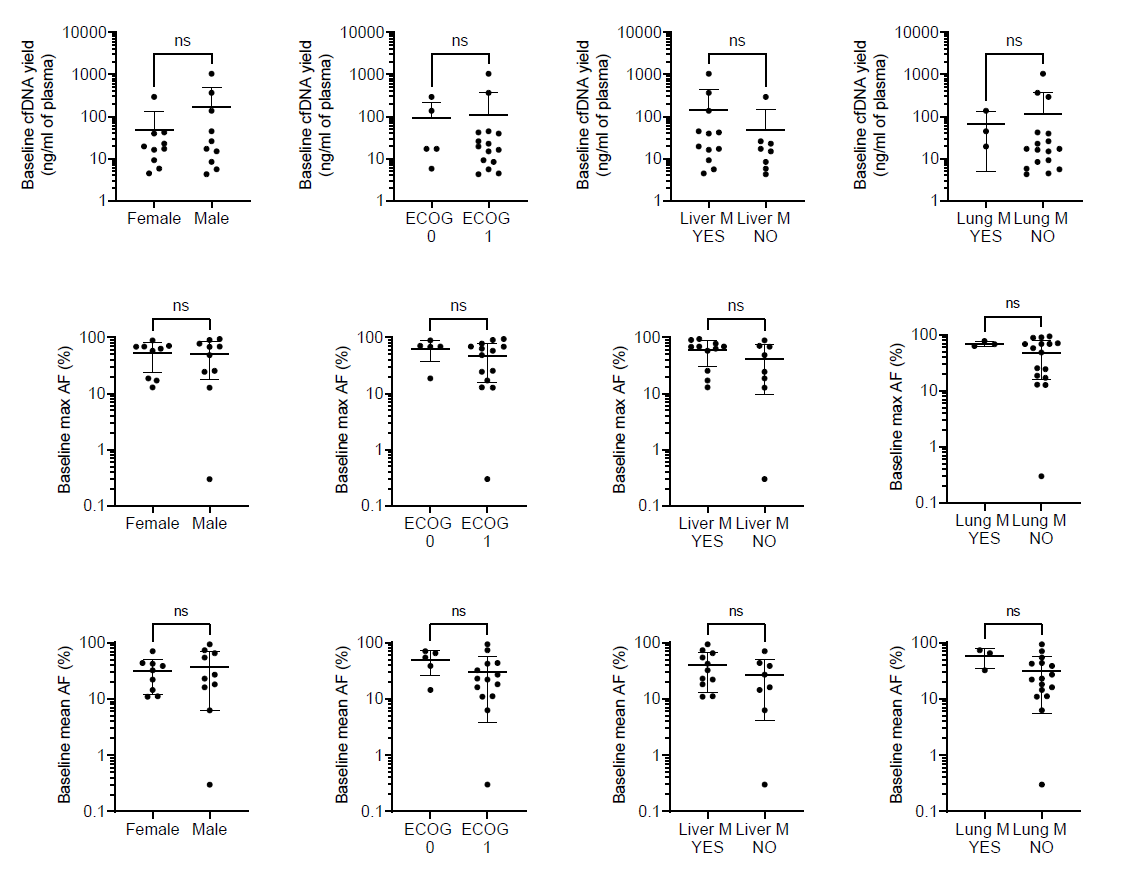


Scatterplots showing distribution of cfDNA levels (top), maximum VAF (middle) and mean VAF (bottom) in patients stratified by gender, ECOG performance status, presence of liver or lung metastases. cfDNA levels shown as ng/mL of plasma used for extraction. Maximum VAF shown as percentage. Mean VAF shown as percentage. All parameters refer to measurements before any treatment. Horizontal lines and error bars indicate mean ± standard deviation. p-values by Mann-Whitney test.

AF, allele frequency; cfDNA, cell-free DNA; ECOG, Eastern Cooperative Oncology Group; M, metastases; ns, not significant; VAF, variant allele frequency.

**Supplementary Figure 7. Association between high baseline CTC level and shorter OS in patients treated with AZD2811**


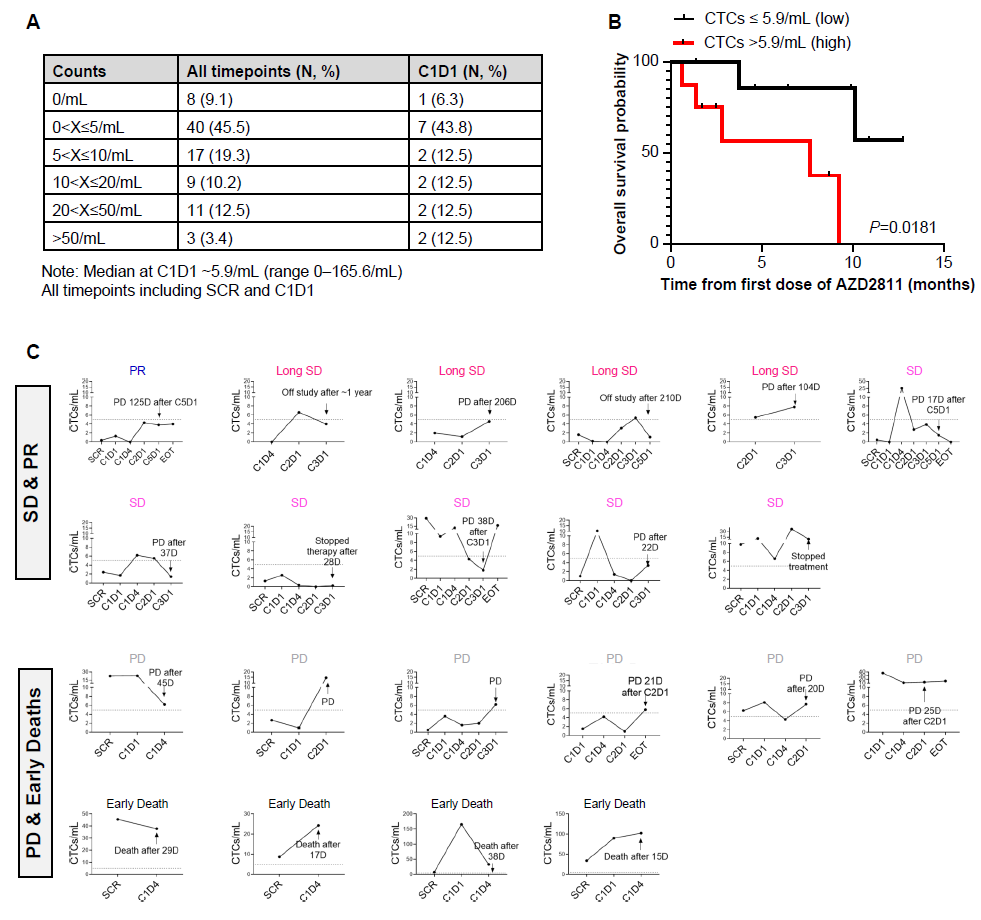


1. Distribution of CTC counts across all timepoints.
2. Kaplan–Meier curves for OS for patients with CTCs below or above the median at C1D1.
3. CTC dynamics in patients treated with AZD2811.

CTC, circulating tumor cell; EOT, end of therapy; Long SD, best objective response of SD and at least two post-baseline SD recorded; OS, overall survival; PD, progressive disease; PR, partial response; SCR, screening; SD, stable disease. Therapy cycles are preceded by the letter ‘C’ and days of the cycle by the letter ‘D’.

**Supplementary Figure 8. Mutation tracking by clinical response**


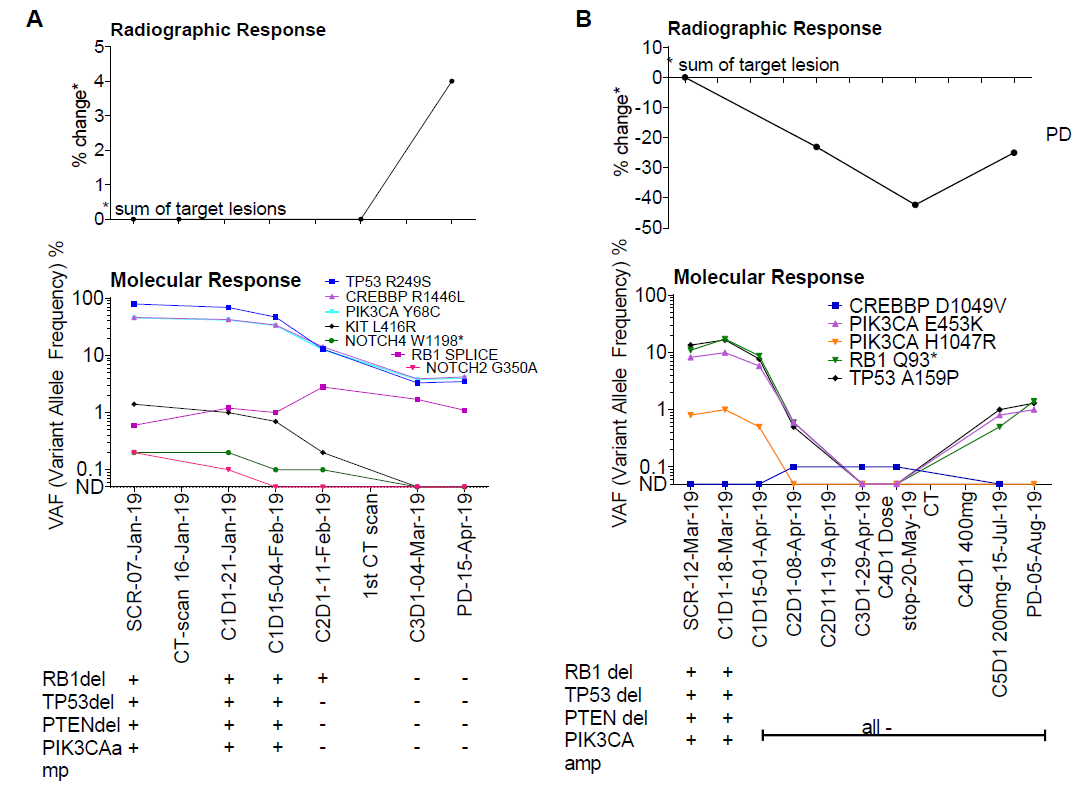


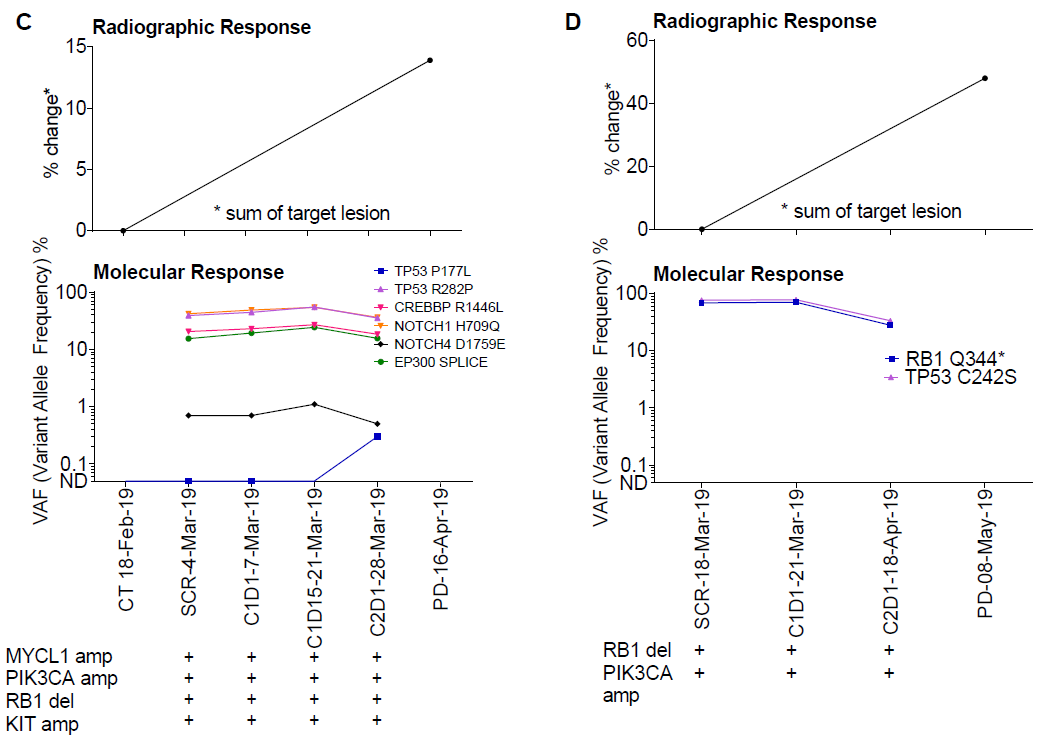


Longitudinal monitoring of mutations in two patients who had SD (A) or a PR (B) following treatment with AZD2811 and two patients who did not experience a PR or SD following treatment with AZD2811 (C, D). The x-axes of both molecular response graphs and radiographic response graphs indicate the sample collection/radiographic assessment timepoints. The y-axes of the molecular response graphs indicate the VAF of the mutations in percentage. Each colored line represents a single mutation in a specific gene identified by the custom targeted panel. The y-axes of the radiographic response graphs indicate the percent change in the sum of target lesions.

CT, computed tomography; EOT, end of therapy; PD, progressive disease; PR, partial response; SD, stable disease; VAF, variant allele frequency. Therapy cycles are preceded by the letter ‘C’ and days of the cycle by the letter ‘D’.

**Supplementary Figure 9. SCLC RNA and protein based subtyping analysis in patients treated with AZD2811**


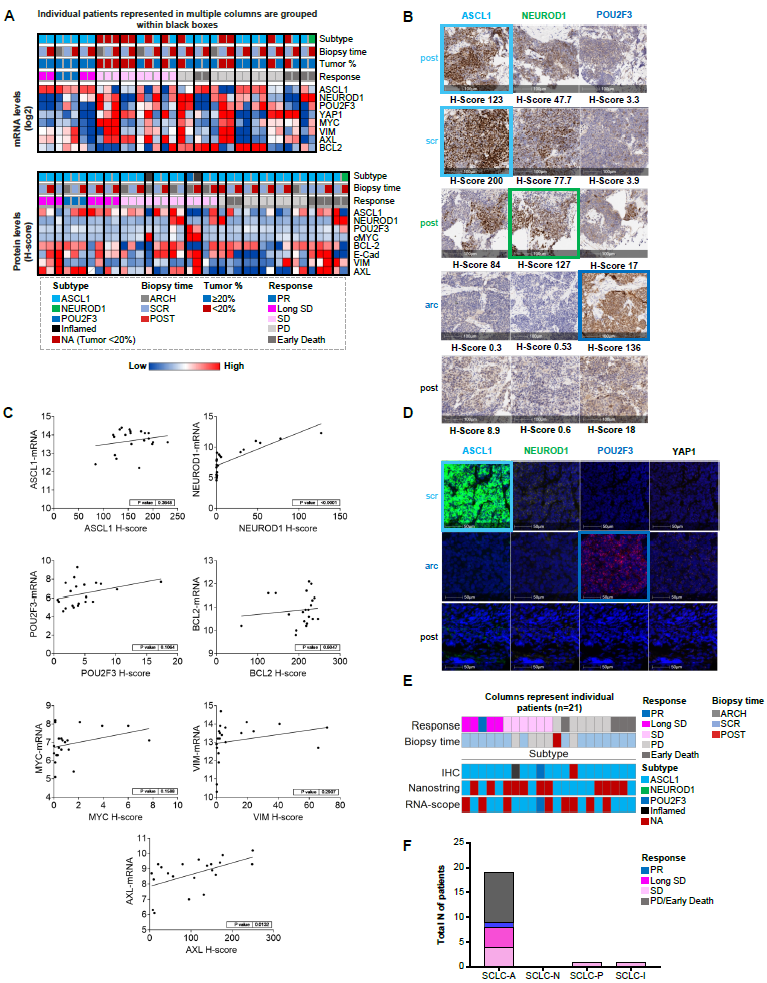


(A) Nanostring-based and IHC-based molecular subtyping of SCLC patients treated with AZD2811 at different timepoints.

(B) Representative IHC images for subtype-defining proteins in SCLC tumor biopsies, color coded based on the subtype (light blue, ASCL1; black, Inflamed; and green, NEUROD1).

(C) Correlation between mRNA and protein expression as detected via IHC in subtype-defining and associated markers.

(D) Representative RNA-scope images for subtype-defining transcription factors in SCLC tumor biopsies, color coded based on the subtype (light blue, ASCL1; black, Inflamed; and dark blue, POU2F3).

(E) and (F) Heatmap and bar graph depicting relationship between baseline subtypes (except for one patient with a post-treatment biopsy) and best overall response to AZD2811 treatment.

ARCH, archival; EOT, end of therapy; IHC, immunohistochemistry; Long SD, best objective response of SD and at least two post-baseline SD recorded; PD, progressive disease; POST, post-treatment; PR, partial response; SCLC, small-cell lung cancer; SCLC-A, ASCL-1 subtype; SCLC-N, NEUROD1 subtype; SCLC-P, POU2F3 subtype; SCLC-I, Inflamed subtype; SCR, screening; SD, stable disease.

**Supplementary Figure 10. Subtype-related molecular features of patients treated with AZD2811**


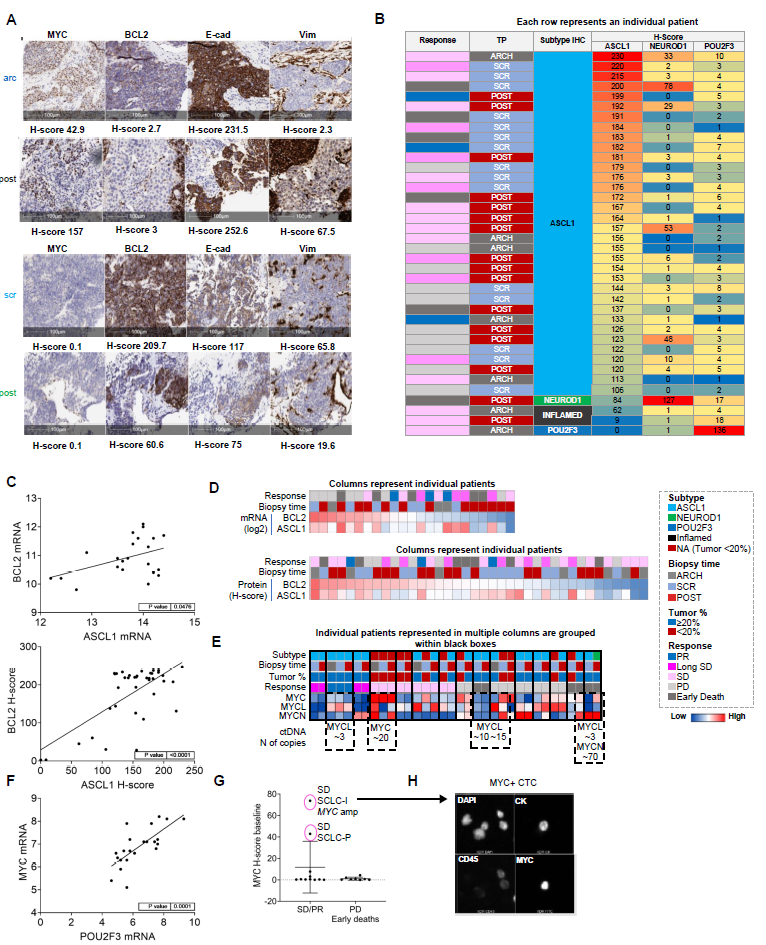


1. Representative IHC images for markers associated with different subtypes (MYC, BCL2, E-cad, and Vim) in SCLC tumor biopsies, color-coded based on the subtype (blue, ASCL1; black, Inflamed; and green, NEUROD1).
2. Heatmap depicting H-scores for ASCL1, NEUROD1 and POU2F2 in tumor biopsies from patients treated with AZD2811.
3. Correlation between mRNA (top) and protein (bottom) levels of BCL2 and ASCL1 in SCLC tumor biopsies.
4. Heatmap depicting ASCL1 and BCL2 mRNA and protein levels (obtained via IHC) in analyzed tumor samples.
5. Heatmap showing mRNA expression levels of MYC family members (MYC, MYCL, MYCN) in SCLC tumor biopsies
6. Correlation between mRNA levels of MYC and POU2F3 in SCLC tumor biopsies.
7. MYC protein levels measured via IHC in patients grouped based on best overall response to AZD2811.
8. Representative image of MYC+ CTC in a patient with SD.

ARCH, archival; CTC, circulating tumor cell; E-cad, E-cadherin; IHC, immunohistochemistry; PD, progressive disease; POST, post-treatment; PR, partial response; SCLC, small-cell lung cancer; SCR, screening; SD, stable disease; Vim, vimentin.**Supplementary Figure 11. Immune features of patients treated with AZD2811**


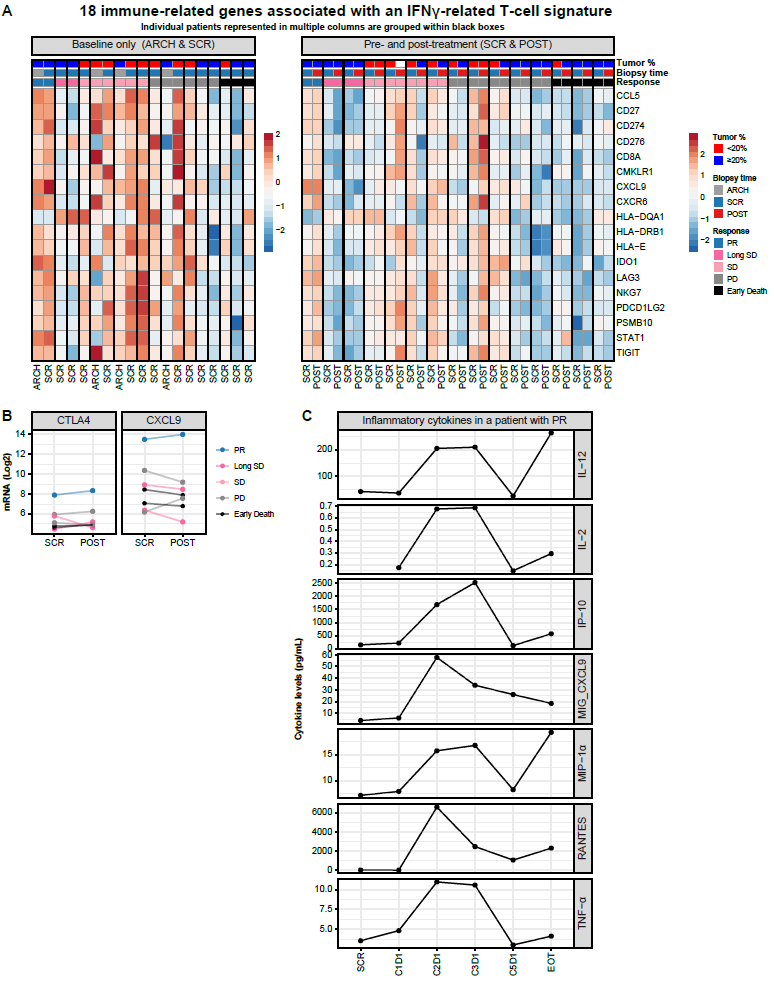


1. Heatmaps comparing 18-gene IFNγ-related T-cell gene expression profile signature (28) across subtyped SCLC tumors. Archival and screening samples are shown on the left, and screening and post-treatment samples are shown on the right.
2. CTLA4 and CXCL9 transcriptomic expression values in screening and post-treatment SCLC samples, color-coded based on best objective response.
3. Plasma levels of inflammatory cytokines in the patient with PR as best objective response.

ARCH, archival; EOT, end of therapy; Long SD, best objective response of SD and at least two post-baseline SD recorded; PD, progressive disease; POST, post-treatment; PR, partial response; SCLC, small-cell lung cancer; SCR, screening; SD, stable disease. Therapy cycles are preceded by the letter ‘C’ and days of the cycle by the letter ‘D’.

**Supplementary Figure 12. Transcriptional features of patients who had a PR or SD following treatment with AZD2811 compared to patients with PD or early death**
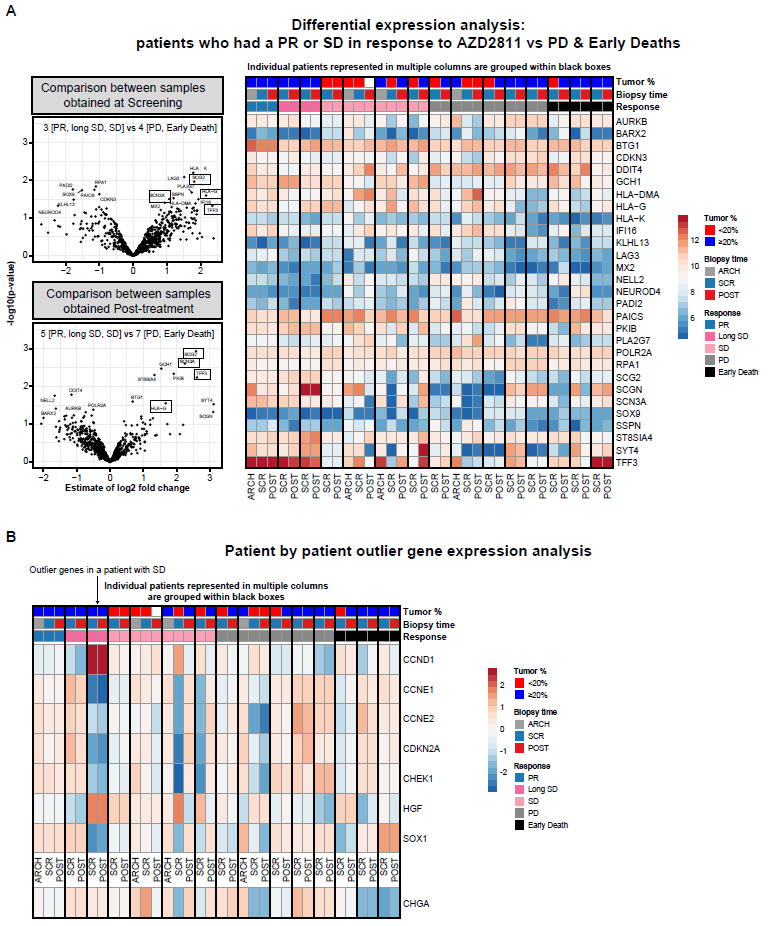


1. Differential expression analysis between [PR, Long SD and SD] and [PD and Early Death] patients at screening and post-treatment (volcano plots on the left) and heatmap of mRNA expression values for highlighted genes (log2 expression values of Nanostring profiling data). Genes with uncorrected p-value <0.05 are named in the volcano plots and included in the heatmap. Genes that appear in the differential expression analysis of both screening and post-treatment samples with uncorrected p-value <0.05 are highlighted within boxes. Samples with less than 20% tumor content as reported by hematoxylin and eosin staining were excluded during the differential expression analysis, but included in the heatmap.
2. Heatmap depicting z-score normalized expression values of the genes identified as outliers for a patient with Long SD vs the rest of the patients (including high expression of *CCND1* and very low expression of *CDKN2A*, *CCNE1* and *SOX1*) and CHGA, a neuroendocrine marker, in 2 out of 3 patients who experienced early death following a patient-by-patient analysis in search of genes whose expression was significantly different from that of the overall cohort.

ARCH, archival; CHGA, chromogranin A; Long SD, best objective response of SD and at least two post-baseline SD recorded; PD, progressive disease; POST, post-treatment; PR, partial response; SCR, screening; SD, stable disease.

**Supplementary Figure 13. Transcriptomic changes detected after AZD2811 treatment**


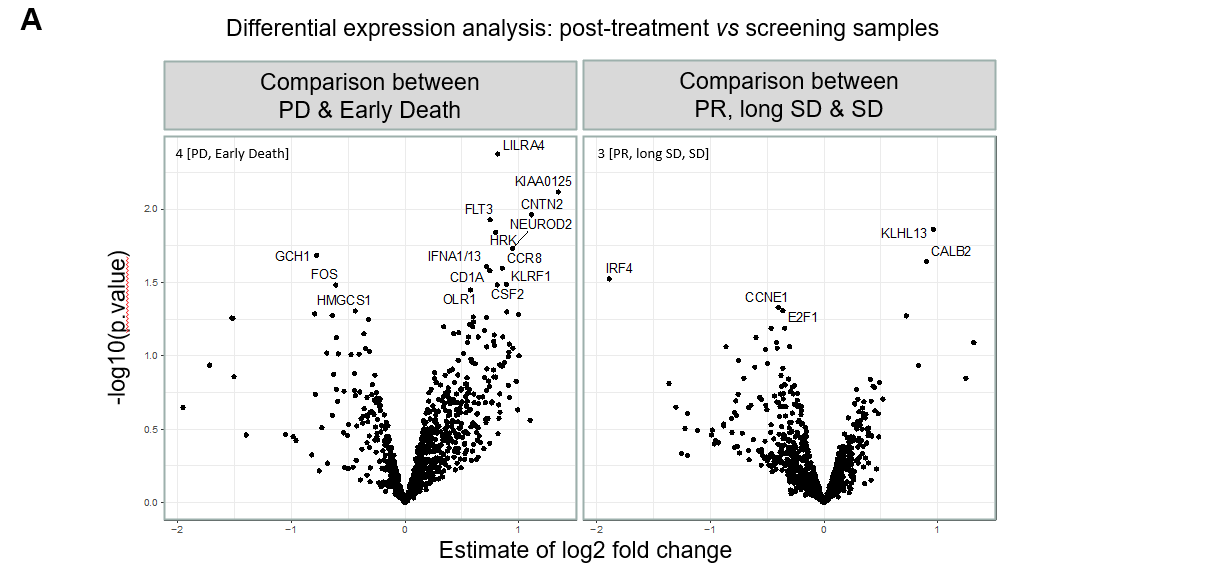


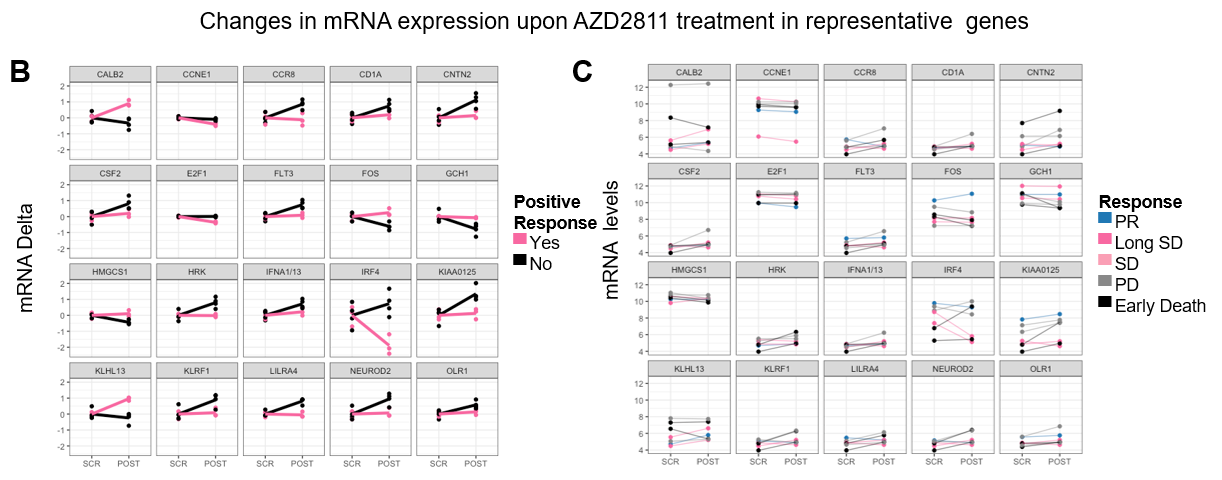


1. Volcano plot depicting differential gene expression of paired post-treatment samples vs screening samples in patients with PD/Early Death (left) and patients with PR/long SD/SD (right). Only samples with more than 20% tumor content as reported by hematoxylin and eosin staining were used for analysis. Genes with uncorrected p-value <0.05 are named in the volcano plots.
2. Visualization of the change in mRNA expression levels of selected genes between the screening and the post-treatment sample fitted by the differential expression analysis. Positive response: Yes = PR, Long SD or SD; No = PD or Early Death.
3. Raw mRNA expression values (log2 expression values of Nanostring profiling data) of selected genes (uncorrected p-value <0.05) at screening and post-treatment. Each line represents one patient color-coded by best objective response.

Long SD, best objective response of SD and at least two post-baseline SD recorded; PD, progressive disease; PR, partial response; SD, stable disease.
